# Harnessing single-cell omics for farmed animal improvement and human health

**DOI:** 10.1186/s40104-026-01516-6

**Published:** 2026-11-10

**Authors:** Houcheng Li, Lingzhao Fang

**Affiliations:** Center for Quantitative Genetics and Genomics, Aarhus University, Aarhus, 8000 Denmark

**Keywords:** Artificial intelligence, Complex traits, Farmed animals, Human diseases, Molecular quantitative trait loci, Population genetics, Single-cell omics

## Abstract

Cellular heterogeneity underlies the regulation of complex traits and diseases, making cell-resolved molecular characterization fundamental for advancing precision agriculture and human biomedicine. Rapid advances in single-cell omics technologies, together with the ever-evolving computational methods, have led to the establishment of large-scale single cell atlases across multiple species, providing unprecedented opportunities to systematically dissect cellular diversity and regulatory mechanisms underlying complex phenotypes. In this review, we summarize recent progresses in single-cell sequencing technologies, reference cell atlas projects, and the integration of single-cell omics with population/quantitative genetics, as well as discuss how artificial intelligence and predictive perturbation modelling are reshaping single-cell research. Finally, we highlight future directions toward population-scale single-cell omics, AI-driven virtual cells/tissues, and genotype–cell/tissue–trait frameworks, which are expected to transform biological discovery, enable better design of veterinary medicines and human therapeutics, as well as accelerate sustainable farmed animal improvement and precision medicine.

## Introduction

Bulk multi-omics sequencing like RNA-seq improves our understanding of biological processes, developmental trajectories, and the genetic basis of complex traits and diseases in both humans and farmed animals. Large-scale projects like the Genotype-Tissue Expression (GTEx) project provide valuable resources for mapping regulatory variants and interpreting the signals of Genome-Wide Association Study (GWAS) for complex diseases in humans [1]. These projects built the foundation for linking genetic variants with disease mechanisms, contributing to the development of precision medicine in humans. Similarly, research in farmed animals has also benefited from bulk multi-omics data. Milestone studies such as the Farm Animal Genotype-Tissue Expression (FarmGTEx) project have greatly enhanced the regulatory annotation of genomic variants in farmed animal genomes [2], including cattle, pigs, sheep, and chickens [3–7]. These efforts improved our ability to explore the regulatory mechanisms underlying complex traits and diseases in farmed animals [8]. However, by averaging molecular signals across heterogeneous cell populations, bulk omics obscure cell-type/state-specific regulatory mechanisms, limiting our ability to accurately resolve cellular heterogeneity, map genetic variation to cellular mechanisms, and decipher the context-dependent regulatory effects that ultimately drive complex traits and diseases.

Single-cell sequencing technologies overcome these limitations by generating high-resolution profiling of gene expression, chromatin accessibility, epigenetic modifications and spatial organization at the individual cell level. In humans, these technologies enhanced our understanding of cellular diversity, disease mechanisms, and tissue microenvironments [9–12]. In farmed animals, single-cell technologies are now being employed to construct comprehensive cell atlases, uncover sex-, breed- and development-specific regulatory programs, map cell-type-specific molecular quantitative trait loci (molQTL), and dissect the genetic basis of complex traits and diseases [13–16]. Although many computational and methodological advances in single cell data analysis were first established in humans, the majority of them are species-independent and therefore readily transferable to farmed animals.

**Fig. 1:**
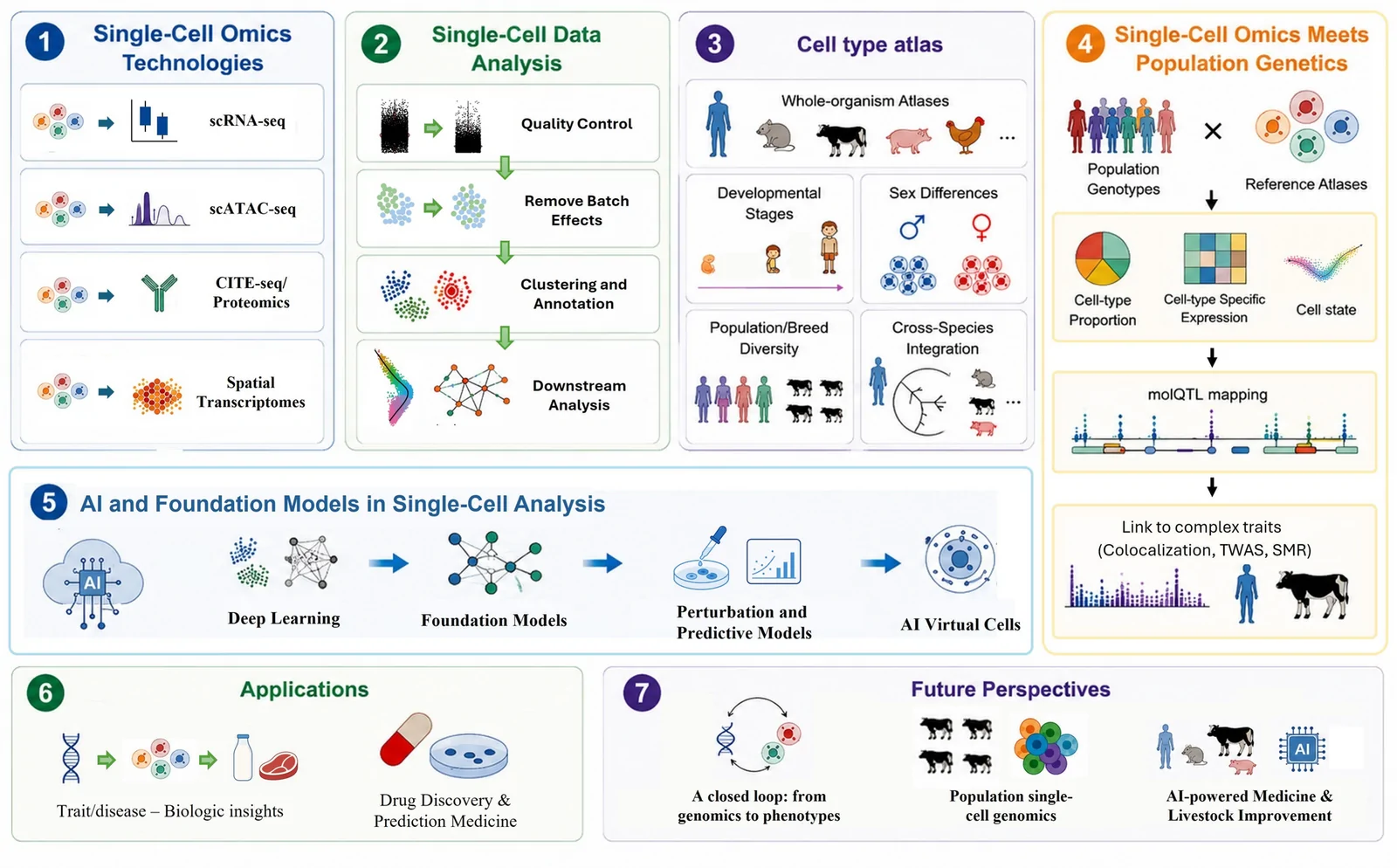
Conceptual overview of the major themes covered in this review. This figure summarizes the major research areas discussed throughout this review, spanning the development of single-cell omics technologies, bioinformatic analysis, reference cell atlas construction, population single-cell genetics, artificial intelligence (AI), applications of single-cell omics in farmed animals and humans, and future perspectives. The panels represent complementary research themes rather than a strictly sequential analytical workflow. **1** Single-cell omics technologies. Representative single-cell technologies provide complementary molecular information at different regulatory layers, including single-cell RNA sequencing (scRNA-seq), single-cell chromatin accessibility profiling (scATAC-seq), CITE-seq/proteomics, and spatial transcriptomics and multi-omics approaches. **2** Single-cell data analysis. Bioinformatic analysis transforms raw sequencing data into biologically interpretable results through quality control, normalization, clustering, cell-type annotation, batch correction and dataset integration, followed by downstream analyses such as trajectory inference, gene regulatory network inference, and cell–cell communication analysis. **3** Cell-type atlases. Large-scale cell atlas projects characterize cellular diversity across whole organisms, developmental stages, sexes, breeds or populations, and species, providing comprehensive reference resources for comparative biology and functional genomics. **4** Single-cell omics meets population genetics. Integration of population-scale genotype data with single-cell reference atlases enables the estimation of cell-type composition, identification of cell-type-specific molecular phenotypes and cell states, molQTL mapping, and integration with genome-wide association studies (GWAS) to identify candidate genes and regulatory mechanisms underlying complex traits. **5** AI-based foundation models in single-cell analysis. Deep learning methods learn informative representations from large-scale single-cell datasets, which can be generalized through foundation models to support predictive modeling, in silico perturbation analysis, and AI virtual cells for simulating cellular responses under different genetic or environmental conditions. **6** Applications. Single-cell omics supports diverse applications, including elucidating disease mechanisms, accelerating drug discovery and precision medicine and facilitating the functional interpretation of economically important traits. **7** Future perspectives. Future developments are expected to focus on closed-loop biology linking genotype, cellular phenotypes and complex traits, population-scale single-cell genomics, cross-species and multi-omics integration, and AI-powered predictive biology to promote both biomedical research and sustainable farmed animal production

In this review, we summarize recent advances in single-cell multi-omics in both humans and farmed animals, highlighting how technological innovations have transformed our ability to characterize cellular diversity and molecular mechanisms underlying complex phenotypes at unprecedented resolution. We first discuss the evolution of single-cell sequencing technologies and large-scale reference atlas projects that provide the foundation for studying cellular heterogeneity across tissues, developmental stages, and even species. We then focus on the emerging integration of single-cell omics with population/quantitative genetics, with particular emphasis on cell-type-specific molQTL, which bridge genetic variation to molecular phenotypes and provide a powerful framework for dissecting the cellular mechanisms underlying complex traits and diseases. Furthermore, we review the rapidly evolving roles of artificial intelligence (AI)-based foundation models in advancing single-cell data analysis from descriptive characterization toward predictive biology. Finally, we discuss how these advances are accelerating disease mechanisms, drug discovery, and genetic improvement in farmed animals, while outlining future challenges and opportunities for population-scale single-cell omics, cross-species integration, and AI-driven biology (Fig. 1).

## Advances in single-cell sequencing technologies

### Evolution of single-cell transcriptome sequencing (scRNA-seq) technologies

**Fig. 2:**
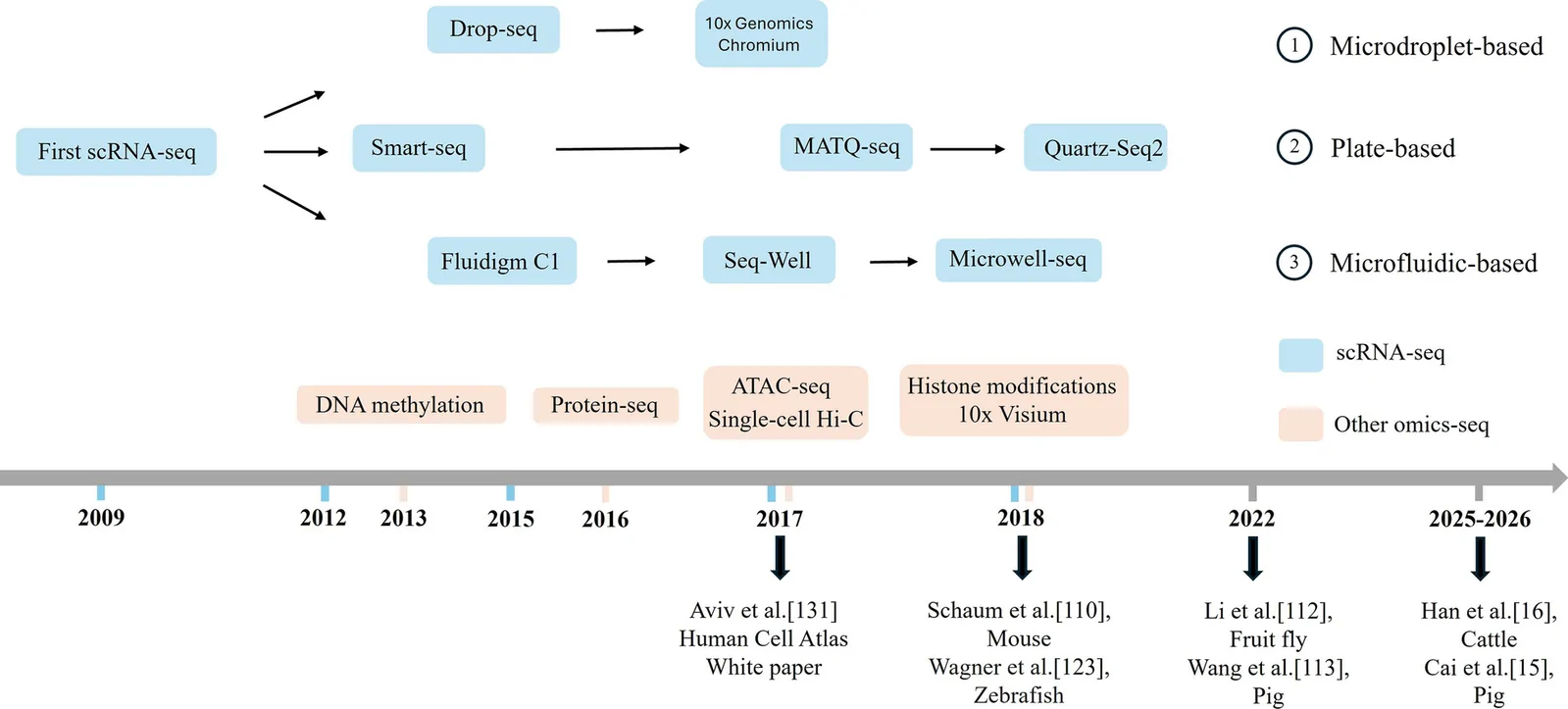
Development of major single-cell omics sequencing techniques and applications. Blue squares represent single-cell transcriptome sequencing (scRNA-seq) techniques and orange one means other omics sequencing technique. scRNA-seq techniques are separated into three categories: plate-based, microdroplet-based and microfluidic-based methods. The timeline at the bottom lists milestone reference cell atlas projects across multiple species

As the earliest developed single-cell sequencing technology, scRNA-seq has become a popular approach for exploring the association between gene expression and complex phenotypes at single-cell resolution. Since the first scRNA-seq protocol was reported in 2009, which used next-generation sequencing (NGS) to amplify and sequence cDNA from individual cells [17], numerous methodological innovations have subsequently expanded the scale and scope of single-cell sequencing, with major technological milestones summarized in Fig. 2. For scRNA-seq, these platforms can be broadly distinguished by their strategies for single-cell isolation and capture:

Plate-based methods (e.g., *Smart-seq*, *Smart-seq2*, *Smart-seq3* and *MATQ-seq*) isolate individual cells into multiwell plates (e.g.*,* 96- or 384-well formats), followed by full-length cDNA synthesis and amplification [18–20]. This design allows near-complete transcript coverage, providing superior sensitivity for detecting low-abundance transcripts, splice isoforms, and allele-specific expression. Such methods are often used in studies requiring deep sequencing of rare cell populations, such as early embryonic lineages or rare immune subsets [21], but their low throughput and high per-cell cost make them less suitable for large-scale surveys.

Microdroplet-based methods (e.g., *10* × *Genomics Chromium*, *Drop-seq* and *Perturb-seq*) use microfluidics to encapsulate individual cells or nuclei together with barcoded beads in oil droplets [22–24]. This approach allows parallel capture of tens of thousands of cells in a single run, making it suitable for large-scale population profiling and discovery of rare cell types. For example, Zheng et al. [22] profiled approximately 68,000 peripheral blood mononuclear cells (PBMCs), demonstrating the scalability of microdroplet-based platforms for large-scale immune cell profiling. However, because sequencing is limited to the 3′ or 5′ end of transcripts, these methods provide lower coverage per gene and are less suitable for isoform-level or alternative splicing analysis.

Microfluidic methods (e.g., *Seq-Well*, *Microwell-seq* and *Fluidigm C1*) separate single cells into microwells or microfluidic chambers, where cell lysis and reverse transcription occur in situ [25–27]. These platforms offer moderate throughput at reduced cost and are suitable for fragile or precious samples, including clinical biopsies.

Altogether, over the past decade, these platforms have improved the evolution of scRNA-seq from a low-throughput approach into a scalable, flexible technology that can be used in diverse experimental goals and biological contexts.

### Technological progress in multi-omics integration

Beyond transcriptomics, advances in single-cell technologies have progressively expanded molecular profiling to epigenomic, proteomic, spatial, and other omics layers (Fig. 2), enabling systematic characterization of diverse molecular phenotypes at single-cell resolution. For instance, single-cell chromatin accessibility sequencing (scATAC-seq) probes open chromatin regions to infer regulatory element activity and transcription factor binding. Multiple platforms such as *10* × *Genomics Chromium ATAC* and high-throughput combinatorial indexing strategies (e.g., *sci-ATAC-seq*) were developed and support parallel profiling of thousands to millions of nuclei [28, 29]. Building on this, single-cell multiome approaches (e.g., *10* × *Genomics Chromium Multiome*) integrate scRNA-seq and single-cell Assay for Transposase-Accessible Chromatin using sequencing (scATAC-seq) from the same cell, allowing the joint analysis of transcriptional programs and *cis*-regulatory landscapes, while methods such as *Paired-seq* extend this integration to include histone modifications [30, 31]. Platforms for single-cell DNA methylome sequencing (e.g., *scBS-seq*, *snmC-seq2* and *scM*&*T-seq*) provide base-resolution maps of DNA methylation, whereas single-cell Hi-C and scChIP-seq reveal 3D genome organization and histone modification patterns, respectively [32–36].

For protein sequencing technologies like CITE-seq and REAP-seq, they combine oligonucleotide-labeled antibodies with scRNA-seq to quantify and link protein abundance and transcriptomes [37, 38]. Spatially resolved single-cell methods, including 10 × Visium, NanoString CosMx SMI and Vizgen MERSCOPE, integrate molecular measurements with intact tissue architecture, retaining critical information on cell–cell interactions and microenvironment [39–41].

More recently, novel single-cell metabolomics and proteomics platforms such as *SCoPE-MS* have begun mapping metabolic and proteomic heterogeneity at the single-cell level, although their throughput and sensitivity currently lag behind transcriptomic and epigenomic assays [42].

### Emerging technologies in single-cell sequencing

Recent advances in single-cell technologies have been driven by the need to characterize cellular and molecular phenotypes at high resolution. First, to enhance resolution and dimensionality, next-generation platforms are moving beyond single-modality transcriptomics toward multi-omics integration within the same cell. For example, 10 × Genomics Chromium Multiome allows joint profiling of gene expression and chromatin accessibility [30], CITE-seq couples RNA-seq with surface protein quantification [37], scGRO-seq captures nascent transcription to resolve transcriptional initiation dynamics [43], while scGET-seq identifies open and heterochromatin states to compute “chromatin velocity” [44]. These approaches expand single-cell analysis from a static molecular state to a dynamic cellular view.

Second, to overcome challenges in specific cell types or tissue microenvironments, specialized protocols have been developed to improve recovery, sensitivity, and specificity. For instance, VINE-seq allows high-purity isolation and single-nucleus RNA sequencing of brain microvascular cells, a cell-type difficult to access with traditional dissociation methods [45], while GEM-X droplet microfluidics increase capture efficiency for low-RNA-content or fragile cells, such as neutrophils [41].

Third, to address targeted biological questions, both new experimental assays and computational methods have been developed to link molecular phenotypes with functional outcomes. For example, DEFND-seq profiles DNA and RNA from the same cell, facilitating reconstruction of clonal evolution and identification of drug resistance mechanisms in tumors [46]. In parallel, computational approaches such as ChromFound leverage large-scale pretrained scATAC-seq data to predict enhancer–gene regulatory relationships across cell types [47].

Overall, these innovations extend single-cell profiling beyond conventional transcriptomic analysis by resolving additional molecular layers, preserving spatial context, characterizing dynamic cellular changes, and, in some cases, linking molecular variation to functional outcomes.

### Data scale and analytical challenges

The rapid development of high-throughput single-cell and multi-omics technologies has resulted in an exponential growth in data scale, covering millions of cells at multiple biological contexts across diverse species. These large-scale datasets lead to significant computational challenges, including storage and processing demands, batch effects arising from technical and biological variability and the need for robust integration across modalities such as transcriptomics, epigenomics, proteomics, and spatial data. Complementing these experimental innovations, integrative computational frameworks (e.g., TotalVI and MultiVI) and foundation models trained on large-scale single-cell datasets are providing scalable solutions for batch correction, cross-modality normalization and integration of space representation [48, 49]. Such methods improve multi-omics from disconnected datasets into coherent, high-dimensional representations of cellular identity and state, facilitating downstream applications such as cell type annotation, trajectory inference, and cross-study meta-analysis. However, further advances in algorithmic scalability, interpretable modeling and multi-scale integration will be important to realize the potential of single-cell omics datasets.

Beyond computational scalability, determining an appropriate sampling strategy has also become an important consideration for large-scale single-cell studies. Unlike bulk transcriptomic experiments, no universal recommendation exists for the number of cells or biological samples required, as experimental design depends on the expected abundance of the target cell populations, sequencing depth, biological variability, and downstream analytical objectives. Recent statistical frameworks provide practical guidance for different scenarios. Probabilistic approaches such as *Howmanycells* estimate the minimum number of cells required to ensure adequate representation of target populations [50], whereas *ncells* further considers marker-gene characteristics and sequencing dropout to estimate the number of cells needed for reliable identification of transcriptionally distinct subpopulations [51]. For studies comparing changes in cell-type abundance across biological conditions, *Sensei* jointly estimates the required numbers of biological samples and cells per sample by accounting for both inter-individual variability and technical sampling uncertainty [52]. These complementary approaches provide practical guidance for designing studies targeting abundant, intermediate-abundance, and rare cell populations while balancing statistical power and sequencing cost. These considerations are particularly relevant for farmed animal studies, where limited sample availability and funding often constrain experimental design.

## Bioinformatic methods in single-cell data analysis

Although development of single-cell technologies has significantly improved the resolution of cellular phenotypes, their potential function can only be explored by using robust bioinformatic methods. Computational advances are important for overcoming challenges such as high dimensionality, sparsity and batch effects, supporting accurate cell type annotation, biologically meaningful analysis and cross-species comparison.

### Bioinformatic analysis for scRNA-seq data

**Fig. 3:**
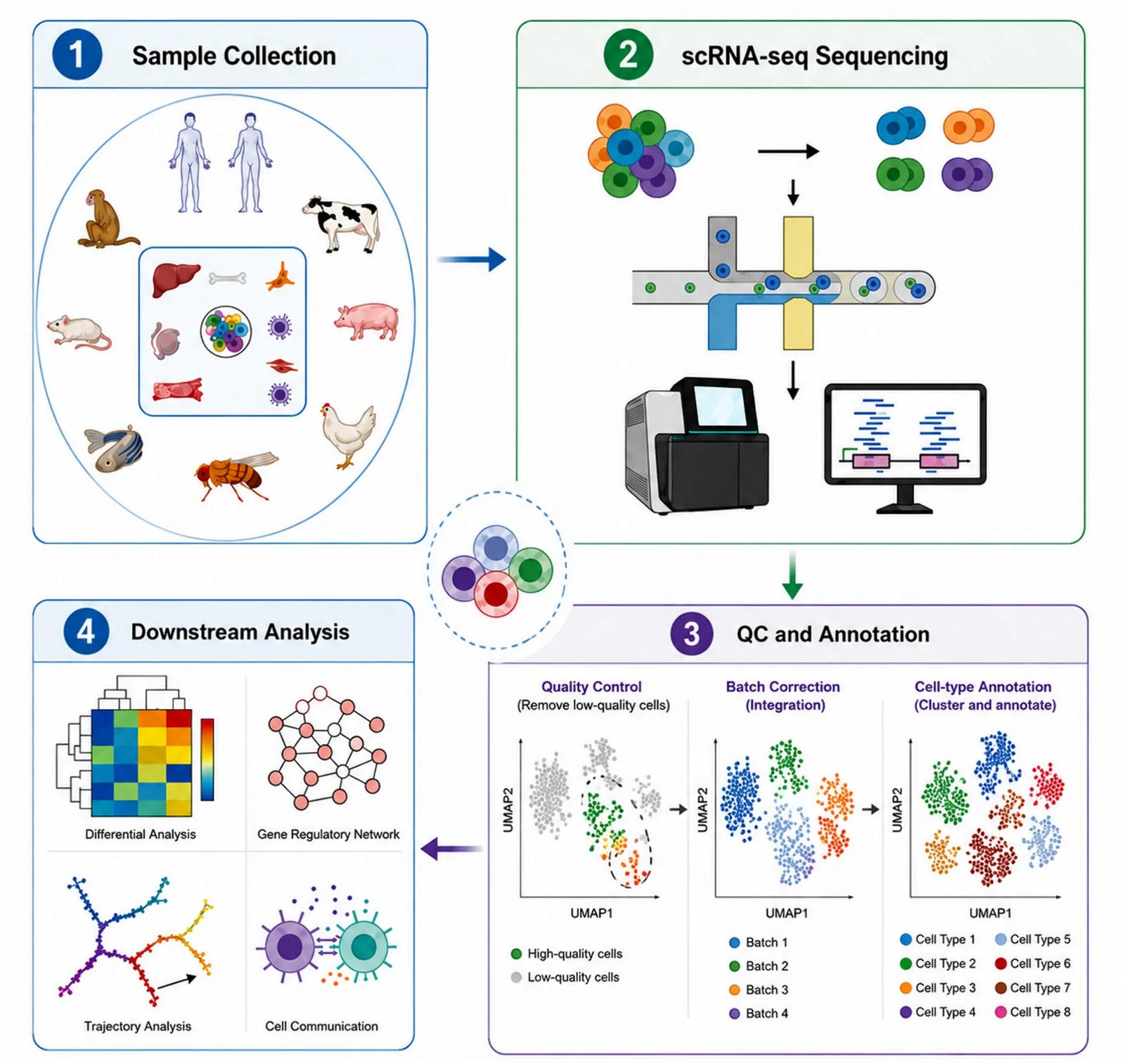
Pipeline of scRNA-seq data analysis. The workflow begins with sample collection from different species and tissues, followed by scRNA-seq sequencing to generate transcriptomic profiles of individual cells. During quality control (QC) and annotation, low-quality cells are first identified and removed (Quality Control), after which batch effects are corrected and datasets are integrated across samples (Batch Correction). The processed cells are subsequently clustered and annotated into biologically meaningful cell types (Cell-type Annotation). The resulting annotated datasets support a wide range of downstream analyses, including differential expression analysis, gene regulatory network inference, trajectory analysis, and cell–cell communication analysis

scRNA-seq is the most popular single-cell omics data type with extensive bioinformatic methods available from upstream data processing and cell annotation to downstream analysis. The major bioinformatic workflow of scRNA-seq analysis is summarized in Fig. 3. Following sequencing, low-quality cells are first removed during quality control, after which datasets are integrated to minimize batch effects before clustering and cell-type annotation, providing a standardized foundation for downstream analyses.

#### Data processing

Single-cell data processing typically starts from raw FASTQ files with initial quality control, including adapter trimming, removal of low-quality bases and filtering of short reads using tools such as FastQC or fastp [53, 54]. For droplet-based platforms (e.g., 10 × Genomics Chromium), cell barcodes and UMIs are extracted to correct amplification bias and remove PCR duplicates. Cleaned reads are then aligned to a reference genome or transcriptome with aligners such as STAR, Cell Ranger or kallisto | bustools, followed by quantification of gene counts per cell [22, 55, 56]. At this stage, reference annotations can retain specific gene biotypes (e.g., protein-coding genes, lncRNAs, or immune receptor genes) relevant to the study [22]. To ensure data reliability, low-quality cells and doublets are filtered using pipelines such as Seurat or Scanpy [30, 57], which apply thresholds on nFeature, nCount, and mitochondrial content. Although effective, these cutoffs are highly dataset- and tissue-dependent, potentially ignoring biologically relevant cells [50]. To overcome this limitation, data-driven approaches such as ddqcR (R package) model the empirical distribution of QC metrics within each cluster, supporting adaptive filtering that better preserves meaningful biological variants [58]. For doublet filtering, several tools are widely used. DoubletFinder (R package) simulates artificial doublets and identifies cells with similar expression profiles [59], Scrublet (Python) employs a nearest-neighbor classifier on simulated doublets to detect multiplets [60] and Solo uses a deep generative neural network for improving sensitivity in complex datasets [61]. Together, these approaches help distinguish true biological cell states from technical artifacts, thereby enhancing clustering accuracy and downstream interpretation. In addition to standard metrics (nFeature, nCount, mitochondrial content and doublets), additional filters are often applied, including ribosomal gene percentage, stress- or dissociation-induced signatures (e.g., FOS, JUN), empty droplets or ambient RNA removal (e.g., EmptyDrops, CellBender) and barcode-based hashing for direct doublet detection [62–64]. These strategies provide finer control of data quality across diverse tissues and protocols.

#### Cell clustering and annotation

Following quality control and filtering, high-quality cells are used in clustering and annotation. Since most single-cell datasets are generated across multiple batches, donors or even platforms, batch effect correction is a crucial preprocessing step to ensure that clusters reflect true biological variation. Methods can be broadly categorized as: anchor-based integration (e.g., Seurat v3/v4) which identifies shared cell populations across datasets to align them [65], graph-based nearest-neighbor approaches (e.g., BBKNN) which construct integrated kNN graphs across batches [66] and latent variable models (e.g., Harmony, LIGER, scVI) which embed cells into shared low-dimensional spaces while regressing out batch-specific variants [67–69]. After correction, dimensionality reduction techniques such as PCA, UMAP or t-SNE are applied, followed by clustering with algorithms like Louvain or Leiden [50]. Cell-type annotation can then be performed using multiple strategies. The most common is marker-based annotation, where cluster-specific genes are manually compared against known lineage markers. Automated reference-based methods, such as SingleR, Garnett and CellTypist, assign cell labels by linking expression profiles with published reference atlases [70–72]. More advanced frameworks integrate multiple modalities, such as scCATCH for tissue-specific annotation or scmap for projection across datasets [73, 74]. Recently, LLM-assisted cell annotation has emerged as a promising strategy for improving automated cell-type annotation. Representative tools include Cell2Sentence [75], which converts gene expression profiles into sentence-like representations for language model learning, scGPT [76], which adapts Transformer-based pretraining for single-cell analysis and cell annotation, and Geneformer [77], which learns contextual gene representations from large-scale transcriptomic data to support diverse downstream tasks, including cell annotation. Together, these approaches improve accurate and efficient mapping of cell-types across diverse biological contexts.

Building on well-annotated single-cell atlases, biological analysis aims to extract functional and mechanistic understanding from cellular populations and their dynamic states. Common downstream analyses can be broadly categorized into the following components:

#### Differential expression and marker gene analysis

Differential expression (DE) analysis is typically the first step after clustering and annotation. By comparing gene expression between clusters, cell-types, conditions or treatment groups, DE identifies marker genes that define specific cell types or states. These markers validate the accuracy of cell type annotations and represent condition-specific changes, such as disease response or metabolism changes. DE related tools, such as Seurat, Scanpy and MAST [30, 57, 78], perform statistical models to correct single-cell data’s sparsity and variability.

#### Pathway and functional enrichment analysis

Gene sets derived from DE analysis are often subjected to pathway enrichment analyses (e.g., Gene Ontology, KEGG, Reactome, and GSEA) to infer functional programs [79–82]. These analyses reveal which biological processes, signaling pathways or molecular functions are enriched in particular cell populations or perturbed under specific conditions. In single-cell data, enrichment analyses can be performed between clusters, cell-types, cell-states or patient subgroups, which provides mechanistic links between gene expression and multiple cellular phenotypes.

#### Trajectory and pseudotime inference

Many biological processes, such as cell differentiation, immune activation or tumor progression, contain continuous transitions rather than discrete cell states. Trajectory inference tools (e.g., Monocle, Slingshot and PAGA) order cells along a pseudotime axis to reconstruct these dynamic processes [83–85]. Such analyses can reveal developmental hierarchies, lineage branching points and transitional intermediate states, offering knowledge into how genetic or environmental perturbations alter developmental trajectories. In addition, RNA velocity-based methods, such as Velocyto [86], scVelo [87] and TIVelo [88], estimate the future transcriptional state of individual cells by quantifying spliced and unspliced mRNA, thereby predicting the direction and speed of cell state transitions. Integrating RNA velocity with trajectory analysis allows detailed annotation of dynamic cell-states as cells progress over real time.

#### Gene regulatory network (GRN) inference

To explore regulators of gene expression changes, GRN inference methods identify transcription factor (TF) and target relationships from single-cell data. Analysis tools, such as SCENIC, CellOracle and GRNBoost, integrate expression profiles with TF motif enrichment to identify active regulatory modules [89–91], identifying key TFs driving specific tissue or cell-type function, biological stages and immune response, thereby linking cellular phenotypes to regulatory mechanisms [92].

#### Cell–cell communication analysis

To catch the cell-to-cell signaling through the expression of ligands, receptors and downstream signaling pathways, computational frameworks such as CellPhoneDB, CellChat, NicheNet and ICELLNET are developed to identify ligand-receptor interactions across cell types and predict communication networks within tissues or disease microenvironments [93–96]. These analyses provide suggestions into how immune cells coordinate responses, how stromal cells support biological balance, and how tumor cells remodel tissue microenvironments, thereby bridging molecular phenotypes with systems-level tissue organization.

### Bioinformatic analysis of other single-cell omics data

Beyond the scRNA-seq data, other single-cell omics data have unique analytical strategies due to their specific data structure and biological questions: 

The scATAC-seq data requires specific processing to identify open chromatin regions, transcription factor binding motifs and *cis*-regulatory elements. Downstream analyses, including peak calling, TF motif enrichment and co-accessibility mapping (e.g., Cicero and ArchR), link distal enhancers to their target genes [97, 98]. For DNA methylome data, analysis focuses on inferring methylation landscapes and cell-type specific epigenetic heterogeneity [32, 33]. Single-cell Hi-C provides 3D chromatin structure and aims to identify cell-type specific chromatin loops, topologically associating domains (TADs) and long-range enhancer–promoter interactions [35]. Together, these analyses reveal the regulation of chromatin architecture underlying transcriptional states in heterogeneous populations. 

In addition, spatially resolved platforms add a tissue-context dimension [39, 41, 99]. Related analyses, such as spatial clustering, cell–cell neighborhood analysis and spatially variable gene identification, connect cellular phenotypes to anatomical microenvironments. Multi-omics extensions, such as spatial proteogenomics, further link spatial transcriptome data with protein [100]. These methods uncover tissue microenvironments, developmental gradients and tumor-immune spatial communication, which are covered in dissociative scRNA-seq. 

As a recently developed field, single-cell metabolomics focus on quantifying metabolic fluxes and metabolite abundances at cellular resolution [101–103]. Analytical strategies include pathway flux reconstruction, integration with scRNA-seq metabolic gene signatures and identification of metabolic heterogeneity across lineages. This is especially relevant for studying cancer, immune activation and stem cell biology, where metabolism plays a defining role in cell fate [104, 105]. 

Although the analytical pipelines differ among individual single-cell omics modalities, integrative analysis across multiple modalities has become increasingly important. Computational integration methods can generally be categorized into paired-data approaches, which jointly analyze multiple modalities measured in the same cells, and unpaired-data approaches, which align independently generated datasets through shared latent representations or corresponding cellular states. For example, TotalVI jointly models paired transcriptomic and proteomic measurements generated by CITE-seq [48], whereas GLUE integrates independently generated scRNA-seq and scATAC-seq datasets through a shared regulatory graph [106]. These examples illustrate that the choice of integration strategy should be guided by experimental design, data availability, and the biological question of interest.

## Single-cell reference atlas projects: system biology across species

The rapid development of single-cell multi-omics technologies, coupled with scalable computational frameworks, has facilitated the establishment of large-scale reference cell atlases across diverse species (Table 1). These projects aim to construct comprehensive maps of cellular diversity and molecular regulation across tissues, sexes, developmental stages, and populations. In both humans and farmed animals, reference single-cell atlas projects have become important resources for systems biology, enabling cross-tissue characterization of cellular heterogeneity and comparative analyses of evolutionarily conserved and species-specific cellular programs. In farmed animals, these atlases also provide opportunities to link genetic variation with cellular mechanisms, complex traits, and local environmental adaptation, thereby bridging fundamental biology with potential applications in animal breeding.

**Table 1:** Single-cell atlas across multiple species

Project	Species	Omics	Cell numbers	Tissue numbers	Category	Published year	Reference
Whole-organism single-cell atlases							
Tabula Sapiens project	Human	RNA	400,000	24	Whole-organism	2022	[107]
Human cell landscape (HCL)	Human	RNA	700,000	60	Whole-organism	2020	[108]
Non-human primates cell atlas (NHPCA)	Macaca fascicularis	RNA	1 million	45	Whole-organism	2022	[109]
Tabula Muris project	Mouse	RNA	100,000	20	Whole-organism	2018	[110]
Mouse Cell Atlas (MCA)	Mouse	RNA	400,000	51	Whole-organism	2018	[111]
Fly Cell Atlas	Fruit fly	RNA	580,000	15	Whole-organism	2022	[112]
Pig Cell Atlas	Pig	RNA	200,000	20	Whole-organism	2022	[113]
Cattle Metabolic Cell Atlas	Cattle	RNA	88,013	10	Whole-organism	2022	[114]
Pig Cell Atlas	Pig	RNA	229,268	19	Whole-organism	2026	[115]
Cattle Cell Atlas	Cattle	RNA	1.3 million	59	Whole-organism	2025	[16]
Buffalo Cell Atlas	Buffalo	RNA	397,011	12	Whole-organism	2025	[116]
Tissue single-cell atlases in Farmed animals							
	Cattle	RNA	6,436	Rumen	Tissue-specific	2021	[117]
	Cattle	RNA, ATAC	30,756	PBMC	Tissue-specific	2022	[118]
	Cattle	RNA	32,708	Skeletal muscle	Tissue-specific	2023	[119]
Context-specific cell atlases							
Human cell landscape (HCL)	Human	RNA	700,000	60	Development-specific	2020	[107]
Fetal human cell atlas	Human	RNA, ATAC	4 million	15	Development-specific	2020	[120]
Mouse Organogenesis Cell Atlas	Mouse	RNA	2 million	Embryos	Development-specific	2019	[121]
Mouse cell differentiation atlas (MCDA) project	Mouse	RNA	520,000	Embryos and 10 tissues	Development-specific	2022	[122]
Zebrafish Developmental Cell Atlas	Zebrafish	RNA	90,000	Embryos	Development-specific	2018	[123]
	Pig	RNA	91,232	Embryos	Development-specific	2024	[124]
	Pig	RNA	2.56 million	Embryos	Development-specific	2026	[15]
	Chicken	RNA	17,628	Limb	Development-specific	2019	[125]
	Human	RNA	419,885	Brain	Sex-specific	2025	[126]
	Human	RNA	27.677	Kidney	Sex-specific	2022	[127]
	Mouse	RNA	2.3 million	17	Sex-specific	2024	[128]
Asian Immune Diversity Atlas (AIDA)	Human	RNA	1,265,624	PBMC	Population-specific	2025	[129]
	Human	RNA	1 million	PBMC	Population-specific	2021	[130]
Buffalo Cell Atlas	Buffalo	RNA	397,011	12	Population-specific	2025	[116]

### Development of whole-organism reference single-cell atlases

Whole-organism single-cell atlases provide comprehensive resources of all major cell types and cell states across multiple organs or tissues. In humans, this concept was first announced by the Human Cell Atlas (HCA) project in 2016, an international collaborative effort to define every human cell type using single-cell multi-omics technologies [131]. Until today, it has already collected 64.4 million cells from 11 thousand donors and built 18 tissue-specific single-cell networks. Building on this vision, the Tabula Sapiens project released in 2022 profiled over 400,000 cells across 24 tissues from multiple donors, supporting cross-tissue comparisons of gene expression and cell type–specific regulatory programs [107]. Two years later, the human cell landscape (HCL) project expanded the human cell atlas with over 700,000 cells from 60 human tissues [108]. The data scale was further expanded in non-human primate cell atlas (NHPCA), where a single-cell transcriptomic atlas of over 1 million cells from 45 tissues of Macaca fascicularis provided an unprecedented resource for investigating tissue composition, cell–cell interactions, infectious disease receptors and human disease orthologues in a species phylogenetically close to humans [109].

At the same time, large-scale whole-organism atlases were generated for model organisms. The Tabula Muris project collected more than 100,000 cells from 20 mouse tissues using both droplet- and plate-based scRNA-seq and generated an atlas controlled for age, environment and epigenetic effects, thereby supporting direct comparisons of cell-type composition within and across tissues [110]. In the same year, the Mouse Cell Atlas (MCA) provided a broader resource, collecting over 0.4 million cells across 51 main tissues in mouse using Microwell-Seq and identifying over 800 cell types and 1,000 sub cell-types [111]. Extending beyond mammals, the Fly Cell Atlas integrated transcriptomic profiles of 580,000 cells from 15 dissected tissues in *Drosophila melanogaster*, offering a powerful reference for investigating genetic perturbations and disease mechanisms in this key model organism [112].

In farmed animals, the first pig whole-organism single-cell atlas was constructed with 20 tissues in 2022 and identified 56 major cell-types to improve the biomedical research in pigs [113]. In the same year, a cross-tissue cell atlas in dairy cattle was reported, identifying 55 cell types across 10 digestive and metabolic tissues and pinpointing specific sub-cell types with nutrients metabolism [114]. Subsequently, the PigGTEx project substantially expanded pig single-cell resources by generating an adult pig atlas comprising 229,268 high-quality nuclei from 19 tissues, annotated into 67 major cell types [115]. More recently, a comprehensive whole-organism single-cell atlas in cattle profiled 1.79 million cells across 59 tissues, identifying 131 cell types and systematically characterizing cell-type specific transcriptional regulation, cellular communication, and heterogeneity. Integration with GWAS analyses further enabled the identification of trait-relevant cell types, providing a valuable resource for functional genomics, precision breeding, and comparative biology [16]. However, compared with human cell atlas projects, current farmed animal studies remain largely focused on individual tissues rather than organism-wide atlases, resulting in uneven tissue coverage and limited cross-study integration. For example, Gao et al. [117] collected scRNA-seq data from 6,436 single cells in cattle rumen and identified six epithelial cell-types, demonstrating their specific functions during weaning. In the next year, they also published the single-cell RNA and ATAC atlas in cattle peripheral blood mononuclear cells (PBMC) with seven cell-types to reveal regulatory mechanism in Lipopolysaccharides (LPS)-driven cell differentiation, gene expression and association of cell-types with milk production traits in cattle [118]. Additionally, Wang et al. [119] built a bovine skeletal muscle single-cell atlas to reveal regulatory mechanisms of intramuscular adipogenesis and fibrogenesis based on a total of 32,708 cells and 21 cell clusters.

Overall, these whole-organism atlas projects have established cross-species cell-type references, supporting comparative analyses of species-conserved and specific cell types, tissue organization, and gene regulatory networks.

### Context-specific cell atlases

Although whole-organism atlases provide comprehensive references of cellular diversity across tissues, context-specific single-cell atlases offer deeper insights into the differences across cell states, compositions and regulatory programs under defined biological or environmental conditions.

Developmental cell atlases capture the dynamic trajectories of cell fate specification, tissue development and lineage branching across time. For example, the human cell landscape (HCL) project (Han et al. [108]) combined scRNA-seq from 60 fetal to adult tissues, supporting comparisons of developmental programs across cell-types and time. In the same year, another study presented a larger human development cell atlas by collecting 4 million cells from 28 samples aging from 72 to 129 days using sci-RNA-seq3, revealing expression changes during the transition from embryonic to fetal stages in mammals [120]. More recently, the Human Developmental Cell Atlas (HDCA) substantially expanded these efforts by integrating single-cell and single-nucleus transcriptomic datasets with spatially resolved multimodal profiling across prenatal human organs, generating a unified atlas spanning 4–22 post-conceptional weeks and comprising approximately 4.6 million cells representing ~ 450 cell types. Beyond cataloguing cell types, the HDCA systematically characterized tissue niches, multicellular interactions, and spatiotemporal regulatory programs underlying organogenesis, providing an unprecedented framework for understanding human prenatal development and the cellular basis of congenital disorders [132]. For model organisms, the Mouse Organogenesis Cell Atlas (Cao et al. [121]) collected over two million cells from embryonic days 9.5–13.5 across multiple organs, inferring lineage relationships and identifying stage-specific progenitor populations. Furthermore, the Mouse Cell Differentiation Atlas (MCDA) project collected over 0.5 million cells from mice across seven developmental stages, spanning from early embryo to mature adult and expanding the mouse development atlas into three new postnatal time points (P0, P10 and P21) [122]. In zebrafish, the Zebrafish Developmental Cell Atlas (Wagner et al. [123]) collected over 90,000 cells from different development stages to reveal cell differences during embryogenesis. For farmed animals, a high-resolution scRNA-seq atlas of pig gastrulation and early organogenesis was constructed with 91,232 cells spanning embryonic days 11.5–15, revealing how temporal and spatial signaling coordinate cell fate determination during early embryonic development [124]. Building upon these early developmental studies, a recent family-based whole-body single-cell atlas profiled 2.56 million single cells and nuclei across 115 maternal and 119 fetal tissues from a pregnant sow and its offspring. This comprehensive resource not only systematically characterized cell identities and tissue-specific transcriptional regulatory programs throughout pregnancy but also uncovered the cellular mechanisms underlying maternal cardiac adaptation and fetal growth restriction, providing an unprecedented framework for studying mammalian development, pregnancy biology, and fetal growth at single-cell resolution [15]. In chickens, a study collected 17,628 cells from three key developmental stages of chicken autopod patterning and identified 23 cell clusters, inferring gene co-expression modules that matched specific tissues during development [125].

Sex-specific atlases reveal the differences in molecular programs and cellular populations between males and females. In human, a total of 419,885 cells from 161 human brain samples, including 72 females and 89 males, were collected and revealed sex differences in brain development at cellular level [126]. Additionally, another study reported sex-based differences in gene expression and metabolism in proximal tubular cells of human kidney [127]. In 2024, a more comprehensive sex-specific study was completed in *Mus musculus*, which constructed a high-dimensional scRNA-seq atlas with over 2.3 million cells from 17 tissues and understood the effects of sex and androgens on the cell-type specific molecular phenotypes [128]. In farmed animals, a recent study established a sex-balanced single-cell atlas across 32 chicken tissues, revealing widespread sex differences in cellular composition and tissue-specific gene expression, particularly in endocrine and immune-related tissues [14].

Population-specific cell atlases help to understand the correlations between population diversity and cellular phenotypes. For example, the Asian Immune Diversity Atlas (AIDA) project collected 1,265,624 circulating immune cells from 7 Asian populations and reported that cellular and molecular phenotypes of immune cells were broadly influenced by sub-continental diversity, ages and sex [129]. In another study, over 1 million cells in PBMC from 222 donors across Central African, West European and East Asian were collected to identify regulatory drivers of population differences in immune responses to SARS-CoV-2 [130]. Recently, the Buffalo Cell Atlas generated a single-cell/nucleus transcriptomic atlas of 397,011 cells from 12 tissues in river and swamp buffalo, identifying 57 cell types and pinpointing metabolic and secretory tissues (e.g., liver, mammary gland and pituitary) and cell types (e.g., hepatocytes, luminal cells, somatotropes and lactotropes) that underlie divergence in milk production [116].

### Multi-species single-cell atlas

Unlike single-species atlases, the cross-species integration provides framework to explore evolutionarily conserved cellular programs and species-specific adaptations, thereby linking evolutionary biology with biomedical and agricultural applications. For example, the non-human primates cell atlas (NHPCA) project compared major cell-types, marker genes and cell communications across three species (monkeys, humans and mice), suggesting species-conserved or specific cellular regulation [109]. Furthermore, cross-species comparison revealed that several complex traits exhibited distinct target-cell specificities across species. For example, migraine-associated variants were more strongly enriched in excitatory neurons in humans and monkeys than in mice, highlighting evolutionary differences in the cellular basis of complex traits [109].

In model organisms, the Mouse Cell Atlas (MCA) project performed a comparative analysis during development stages in seven species with different evolutionary distances at single-cell resolution and found regulatory programs in common cell-types that are broadly active during specific development stages of invertebrates and vertebrates [111]. In the next year, they further constructed a cross-species cell atlas and identified molecular, cellular phenotypes and biologic pathways that changed throughout the longevity by integrating more than 2.6 million single cells from three species (mice, zebrafish and *Drosophila*), revealing the conservation and diversity of cell-types at genetic and regulatory levels [133].

For non-model species, a cross-species single-cell atlas was built with 11 species (cat, dog, hamster, lizard, goat, rabbit, duck, pigeon, pangolin, tiger and deer) [134]. Cross-species analyses uncover shared developmental programs, key cellular networks and conserved regulatory pathways across these evolutionarily distant species, while also pointing to potential SARS-CoV-2 target cell types and possible zoonotic reservoirs. In farmed animals, the pig whole-organism single-cell atlas compared the cell–cell communication from endothelial cells between pig and human and found specific active communications between endothelial cells and other cell types in pigs [113]. Another study in farmed animals combined scRNA-seq and scATAC-seq to characterize regulatory mechanism of somatic cells and primordial germ cells (PGCs) in early gonads of goats, pigs, macaques and humans, understanding the evolution and development of PGCs and gonadal somatic cell in mammals [135].

Although most comprehensive single-cell atlases have been generated in humans and model organisms, the experimental and computational frameworks established by these projects—including standardized tissue processing, batch correction, cell-type annotation, atlas integration, and cross-dataset harmonization—are easily transferable to farmed animals. The current limitation for farmed animals therefore lies primarily in the availability of sufficiently large and diverse reference cell datasets rather than in methodological development.

## When single-cell omics meet population/quantitative genetics

Based on large-scale single-cell atlas projects, the integration with population genetics supports the mapping of genetic variants to specific cell types and regulatory mechanisms, linking population-level statistical associations of complex traits, such as from GWAS and selection signatures, with genetic regulation at cellular level.

### Deconvolution-based molecular phenotypes at cellular level

The core of integrating single-cell omics with population genetics is defining molecular phenotypes at cellular level. In the earlier studies, high expenses of single-cell omics sequencing limited sample sizes. As an alternative approach, deconvolution-based methods were developed to infer cellular information (e.g., cell-type composition or gene expression) in bulk RNA-seq samples using single-cell references, providing a cost-efficient strategy to capture cellular heterogeneity for downstream analyses such as expression QTL (eQTL) mapping and trait association.

Among the phenotypes estimated by deconvolution, cell-type composition serves as the foundation for inferring other cellular phenotypes. Several deconvolution strategies have been developed and broadly categorized into three groups: reference-based, marker gene-based and reference-free approaches. Reference-based methods require a predefined reference matrix that includes all gene expression values of each cell type (e.g., MuSiC [136], SCDC [137] and DWLS [138]). Marker gene-based approaches rely on the expression profiles of a selected set of marker genes known to exhibit differential expression across cell types (e.g., Cibersort [139] and DeconRNASeq [140]). On the other hand, reference-free methods do not necessitate reference profiles or they may only need the identities (names) of cell-type specific genes (e.g., CDSeq [141], TOAST [142] and BayesCCE [143]), offering flexibility in the absence of comprehensive single-cell reference. Recent benchmarking studies have shown that no single deconvolution method consistently outperforms all others across tissues and experimental settings. Instead, deconvolution accuracy is jointly determined by multiple factors, including the quality of the reference atlas, marker gene selection, tissue complexity, sequencing protocol, and the presence of unknown cell types. Reference-based methods generally achieve the highest accuracy when well-matched single-cell references are available, whereas reference-free approaches provide greater flexibility for poorly characterized tissues but often at the expense of precision. Furthermore, benchmarking studies consistently demonstrate that maintaining gene expression in a linear scale and using complete, high-quality reference signatures have a larger impact on performance than the choice of deconvolution algorithm itself, highlighting that careful reference construction is often more critical than selecting a specific computational method [144–147]. Therefore, the choice of deconvolution method should be guided by the biological question and available reference data rather than by a universal performance ranking. Recently, methods such as MeDuSa [148] have extended deconvolution beyond cell types to the cell-state level, using mixed models and scRNA-seq references to estimate state abundances along trajectories in bulk data.

Building on composition estimates, deconvolution frameworks can also infer cell-type or cell-state specific gene expression profiles. Among these, Bayesian MIND (bMIND) has been developed as a widely used and robust approach, combining prior information to regularize estimation in this statistically challenging setting [149]. BayesPrism extends this concept with a hierarchical Bayesian framework and infers both cell-type proportions and gene expression with reduced reference bias [150]. DESCEND, as a distribution-based approach, recovers full gene expression distributions within each cell type beyond mean levels and captures higher-order features such as variability and Gini indices [151].

In summary, deconvolution-based approaches provide a scalable and cost-effective bridge between single-cell references and population-scale bulk data, enabling the estimation of diverse cellular phenotypes that serve as critical foundations for regulatory mapping and trait dissection. In addition, because the underlying computational principles of deconvolution are largely species independent, many analytical frameworks originally developed for human datasets can be adapted for use in farmed animals when appropriate species-specific reference atlases are available [136, 138, 139]. Their performance in farmed animals is expected to depend primarily on the availability and quality of tissue-matched single-cell reference atlases rather than on species-specific algorithmic modifications.

### Population-based molecular phenotypes at cellular level

Although deconvolution-based methods extend single-cell information to population-scale bulk samples in a low-cost manner, they still rely on the quality of single-cell references and are not accurate enough to estimate the cellular phenotypes in rare cell-types [144, 147]. In contrast, direct measurement of molecular phenotypes (e.g., gene expression, splicing, chromatin accessibility, and epigenetic modifications) using population-scale single-cell sequencing enables direct characterization of rare cell types and dynamic cell states while providing cell-type-resolved molecular phenotypes [131]. As sequencing costs decrease, these datasets are regarded as the gold standard for defining cellular and molecular phenotypes in population genetics and are widely used to establish single-cell eQTL, splicing QTL (sQTL) and other molecular QTL directly from single-cell omics data.

In scRNA-seq, molecular phenotypes can be defined at multiple levels. By aggregating cells of the same cell-type, pseudo-bulk expression profiles can be constructed at the cell-type level to increase the statistical power. Droplet and microwell-based scRNA-seq platforms (e.g., 10 × Genomics Chromium, Drop-seq and Microwell-seq) support the identification of alternative polyadenylation (APA) and the usage of different polyadenylation sites that generate variable 3′UTR lengths [22, 23, 26]. Plate-based methods (e.g., Smart-seq) capture full-length transcripts, allowing the identification of diverse molecular phenotypes similar to bulk RNA-seq, such as alternative splicing (different exon combinations generating multiple isoforms), exon expression (differential exon usage), enhancer activity (transcriptional regulation of non-coding elements) and isoform expression (quantification of different transcripts generated by splicing events) [18].

In single-cell epigenomics, a wide range of molecular phenotypes can also be defined. Chromatin accessibility, the openness of chromatin to transcription factors, can be captured by scATAC-seq [152]. DNA methylation, which regulates gene expression through cytosine modifications, can be profiled at single-cell resolution using methods such as scBS-seq, scRRBS, snmC-seq2 and scM&T-seq [32–34, 153]. Histone modifications (e.g., H3K27ac, H3K4me3), defining active or repressed chromatin states, can be identified with scChIP-seq or CUT&Tag [154, 155]. 3D chromatin architecture, including loops and topologically associating domains (TADs), can be mapped by scHi-C [156]. In addition to transcriptomics and epigenomics, protein abundance can be quantified using technologies such as CITE-seq and SCoPE-MS [37, 42].

In summary, population-based single-cell sequencing directly captures a broad range of molecular phenotypes across transcriptomic, epigenomic, proteomic, and metabolic layers, providing an unparalleled resolution for studying gene regulation and complex traits [157]. Compared with deconvolution-based approaches, direct population-scale single-cell sequencing provides direct and cell-resolved measurements of cellular molecular phenotypes, enables the discovery of rare cell types and dynamic cell states, and extends analyses beyond transcriptomics to additional molecular layers [131]. These advantages make it an important framework for future population genetics and integrative multi-omics studies, although its widespread application is still constrained by sequencing costs and limited sample sizes. As sequencing costs continue to decline, these population-scale single-cell strategies are expected to become increasingly applicable to farmed animal populations, particularly for FarmGTEx populations where large bulk RNA-seq cohorts already exist [13].

### Single-cell molQTL

Building on cell-type-resolved molecular phenotypes, mapping cell-type-specific molecular quantitative trait loci (molQTL) has become a powerful strategy for investigating how genetic variants influence molecular regulation in specific cellular contexts. Major single-cell molQTL studies in humans and farmed animals are summarized in Table 2. Among these, single-cell expression QTL (eQTL) studies are currently the most extensively investigated, supported by well-established analytical frameworks and the widespread application of scRNA-seq technologies. Two major strategies have been commonly used for cell-type-specific eQTL mapping: deconvolution-based approaches and direct population-scale single-cell analyses.

**Table 2:** Major single-cell molQTL studies in humans and farmed animals

molQTL/Gene number	Specie	Tissue/cell-type	Category	Published time	Reference
1,988 eGenes	Human	Lymphoblastoid cells	Population	2013	[158]
26,597 independent *cis*-eQTL, 990 *trans*-eQTL	Human	PBMCs	Population	2022	[159]
6,511 eGenes	Human	T cells	Population	2022	[11]
25,273 eQTL	Human	Brain	Deconvolution	2024	[160]
4,629 eGenes	Human	Blood	Deconvolution	2024	[161]
3,347 ieGenes, 987 isGenes	Human	35 tissues, 7 cell-types	Deconvolution	2020	[12]
2,326 ieGenes	Human	Kidney	Deconvolution	2021	[162]
376 ieGenes	Pig	3 tissues	Deconvolution	2024	[4]
128 ieGenes	Chicken	6 tissues	Deconvolution	2025	[5]
16,295 eGenes 16,341 cell-type ieGenes 14,328 cell-state ieGenes	Cattle	22 tissues	Deconvolution	2025	[13]
9,628 ATAC-QTL	Human	Prefrontal Cortexes	Population	2023	[163]
Hundreds of thousands meQTL	Human	PBMC	Population	2024	[164]

Deconvolution-based strategies extend single-cell information to population-scale bulk datasets in a low-cost manner. By using bulk RNA-seq together with single-cell references, these methods infer cellular information across large-scale populations, supporting downstream molQTL mapping. For example, Dai et al. [160] identified 25,273 cell-type eQTL from 1,112 bulk RNA-seq samples with the deconvolution strategy in the ROSMAP cohort. Similarly, a study on neuropsychiatric disorders identified 4,629 cell-type eGenes from 1,730 whole-blood RNA-seq samples, including 1,211 absent from bulk tissue analyses [161]. In addition to expression, deconvolution can also estimate cell-type and cell-state fractions and support interaction eQTL (ieQTL) mapping, which detects the interaction effects between genotype and cell composition. Using GTEx data, Kim-Hellmuth et al. [12] identified 3,347 ieQTL and 987 interaction splicing QTL (isQTL) across 35 tissues, extending eQTL analyses to organism-wide cellular resolution. In 2021, Sheng et al. [162] collected 659 human kidney samples and identified 1,613 and 713 cell-type ieQTL in tubular and glomerular samples respectively, suggesting the importance of proximal tubules for kidney function and endothelial cells and distal tubule segments for blood pressure pathogenesis. In farmed animals, the FarmGTEx project also reported ieQTL across species, such as 376 significant ieGenes in three pig tissues and an average of 129 ieGenes across six chicken tissues [4, 5]. Building upon these studies, another PigGTEx study substantially expanded cell-type specific regulatory mapping by integrating 3,921 bulk RNA-seq samples with a large-scale single-cell atlas from 19 pig tissues, identifying thousands of ieQTL that provided cell-type specific insights into 268 complex traits through GWAS colocalization [115]. Recently, a comprehensive cattle cell-type regulatory atlas within the FarmGTEx project substantially expanded these efforts by integrating 8,364 bulk RNA-seq samples with 999,192 single cells representing 81 cell types across 22 tissues. Through joint mapping of cell-type specific eQTL and cell-type/state interaction eQTL, together with bulk eQTL colocalization, the study identified 57,043 novel cell-resolved regulatory variants affecting 18,153 genes, demonstrating markedly greater tissue and cell-type specificity than conventional bulk eQTL. Furthermore, integration with GWAS for 44 complex traits revealed 505 additional trait-associated loci that were undetectable using bulk eQTL alone, while combining these regulatory variants with artificial selection signatures provided cell-type specific insights into the genetic architecture of economically important traits and precision breeding. As the first large-scale cell-resolved regulatory variant atlas in cattle, this resource provides a comprehensive framework for interpreting the cellular mechanisms underlying complex traits, evolution, and genomic selection in farmed animals [13].

Although deconvolution-based methods provide a low-cost framework, they rely on the quality of available single-cell references. Rare cell types may be missed and subtle regulatory variants within heterogeneous populations can remain undetected. These limitations motivate another strategy: direct population-scale single-cell sequencing. The single-cell population-based strategy captures molecular phenotypes (e.g., gene expression, alternative splicing and alternative polyadenylation) at single-cell populations and directly associates them with genetic variants. Early work in 2013 by Wills et al. [158] collected 1,440 lymphoblastoid cells from 15 individuals, suggesting the association of genetic variants with molecular phenotypes at cellular level. However, the limited sample size constrained discovery power. A later study in 2022 analyzed scRNA-seq data from 1.27 million PBMCs across 982 individuals, identifying 26,597 independent *cis*-eQTL and 990 *trans*-eQTL across 14 cell types. This work revealed dynamic allelic effects in B cell differentiation from naïve to memory states, highlighting that common variants drive interindividual variants in immune function [159]. More recently, Nathan et al. [11] improved the resolution from cell types to cell states, collecting over 500,000 memory T cells from 259 individuals and discovering 6,511 cell state-dependent *cis*-eGenes. They demonstrated that continuous cell states explained more eQTL than discrete categories, highlighting the value of cell-state resolution for dissecting gene regulation in functionally heterogeneous cell populations. In addition to transcriptomics, epigenomic QTL have also been mapped at the single-cell level. Xiong et al. [163] generated a multi-omics atlas of 850,000 nuclei from 92 human prefrontal cortices using snRNA-seq and snATAC-seq, identifying 9,628 cell-type specific ATAC-QTL and revealing Alzheimer’s disease-associated regulatory mechanisms. At the same time, a hierarchical Bayesian interaction (HBI) framework inferred hundreds of thousands of cell-type specific methylation QTL (meQTL) across six immune cell types from bulk data, linking genetic variants to immune regulatory functions [164]. Collectively, deconvolution-based and population-based strategies should be regarded as complementary rather than competing approaches. The former maximizes statistical power in large cohorts, whereas the latter provides the highest cellular resolution for mechanistic discovery. Although the general statistical principles of molQTL mapping can be applied across species, their implementation must be tailored to the genetic architecture of the target population. In farmed animals, particular attention should be paid to population structure, including family relatedness, breed stratification, linkage disequilibrium patterns, and admixture history, as these factors can substantially influence association signals and statistical inference [2, 165].

### Statistical models for single-cell molQTL mapping

For molQTL mapping, the choice of statistical model is important. Linear regression models (LMs) remain the most widely used framework for aggregating cellular molecular phenotypes into pseudo-bulk profiles because of its efficiency, interpretability, ability to scale to large datasets and flexibility to include covariates for confounder control. After normalization, many molecular phenotypes in pseudo-bulk profiles approximate a normal distribution, making them suitable for LMs. Widely used software packages such as FastQTL, tensorQTL and QTLtools have been developed based on this framework [166–168]. Building on LMs, linear mixed models (LMMs) further add random effects to account for population structure, relatedness and batch effects, thereby reducing confounding and enhancing statistical power. Importantly, LMMs are particularly advantageous in farmed animal populations, where complex pedigrees, strong population stratification and shared environmental influences lead to major challenges for molQTL detection [169].

Nevertheless, regression strategies based on pseudo-bulk aggregation assume homogeneity within the same cell-type and often ignore the heterogeneity from cell states or cell cycle stages. To overcome this limitation, some studies have directly detected molecular phenotypes across single-cell populations. Unlike pseudo-bulk profiles, the single-cell molecular phenotypes under population level are extremely large and sparse and not suitable for LMs or LMMs. Recent studies have shown that Poisson and negative binomial models are particularly suitable to scRNA-seq data measured at population scale, as they directly model count data and naturally accommodate both the high frequency of zeros and overdispersion [11].

In addition, interaction models have been developed to estimate cell-type fraction effects in bulk molQTL mapping. These models use an interaction factor between genotype and estimated cell-type fractions, thereby identifying interaction molQTL whose effects are enriched in specific cell-types. For example, in bulk eQTL studies, association of genotypes with gene expression may depend on the abundance of the relevant cell type, uncovering cell-type specific regulation masked in bulk data. By using single-cell resolution to deconvolute cell-type specific effects, interaction models provide a powerful framework for studying genetic regulation in heterogeneous tissues [12, 162].

Overall, molQTL mapping has evolved from traditional linear regression models toward more sophisticated statistical frameworks, each addressing distinct analytical challenges. Linear regression models remain the preferred choice for pseudo-bulk analyses because of their computational efficiency, scalability, and interpretability [166–168]. Linear mixed models further improve robustness by accounting for population structure and family relatedness, making them particularly suitable for human and farmed animal populations with complex pedigrees [169]. Count-based models such as Poisson and negative binomial regression are better suited for population-scale single-cell data, where sparse and overdispersed expression profiles violate the assumptions of linear models [11]. Interaction models provide an additional layer of resolution by explicitly modeling genotype–cell composition interactions, enabling the identification of context-dependent regulatory effects that are masked in conventional bulk analyses [12]. Rather than representing competing approaches, these statistical models are complementary, and their optimal application depends on the molecular phenotype, data structure, and biological question being addressed.

## Artificial intelligence and foundation models in single-cell analysis

As single-cell datasets continue to expand in both scale and complexity, traditional analytical frameworks face growing challenges in scalability, multimodal integration, and biological interpretation. To address these limitations, artificial intelligence (AI) and foundation models have emerged as transformative tools, enabling automated cell type annotation, cross-omics integration, trajectory inference, and predictive modeling of cellular states. These approaches extend beyond conventional bioinformatics by leveraging deep learning and large language models to capture high-dimensional patterns and incorporate prior biological knowledge, thus opening new avenues for single-cell analysis.

### Deep learning models for single-cell data

**Fig. 4:**
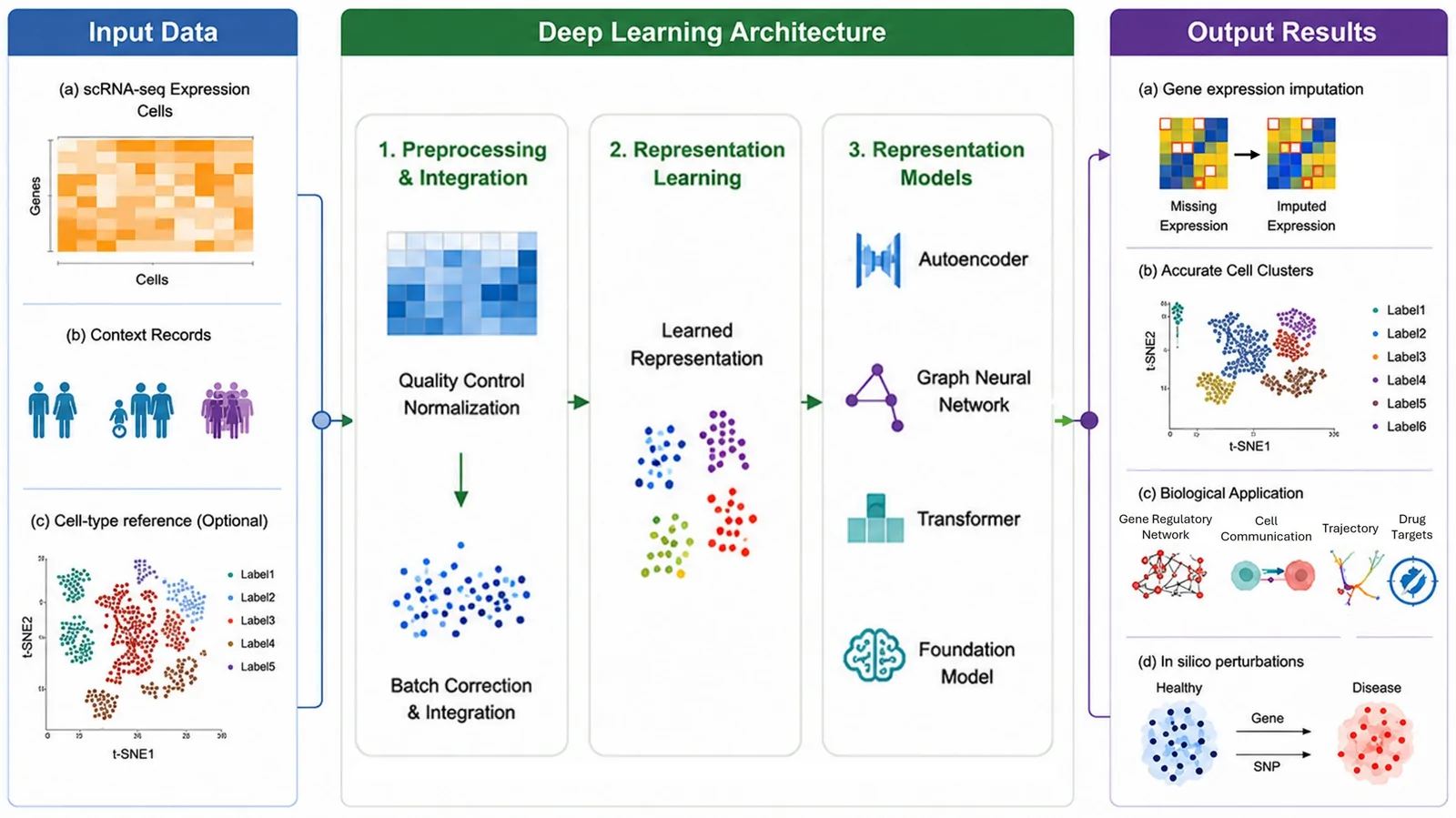
Deep learning framework in predicting single-cell information. The figure summarizes the general workflow of deep learning for single-cell omics analysis, from data preprocessing and representation learning to model training and downstream biological applications. Input Data, three major sources of information can be used for model development: (**a**) single-cell RNA sequencing (scRNA-seq) expression matrices, which provide the primary molecular features; (**b**) contextual information, including donor characteristics, disease status, treatment conditions, developmental stage, or other sample-level metadata; and (**c**) optional cell-type reference datasets, which can support supervised learning, transfer learning, and cell-type annotation. Deep Learning Architecture, the analytical workflow consists of four sequential stages. **1.** Preprocessing and integration perform quality control, normalization, batch correction, and dataset integration to generate high-quality input data. **2**. Representation learning compresses high-dimensional molecular profiles into informative latent representations that preserve biological variation while reducing technical noise. **3**. Representation models include four representative deep learning architectures discussed in this review: autoencoders, graph neural networks (GNNs), Transformer-based models, and foundation models. Output Results, the trained models support a broad range of biological applications, including (**a**) gene expression imputation, (**b**) improved clustering and cell-type annotation, (**c**) downstream biological analyses such as gene regulatory network inference, cell–cell communication analysis, trajectory inference, and therapeutic target discovery, and (**d**) in silico perturbation analyses to predict the effects of genetic or molecular perturbations on cellular states. Blue arrows indicate the overall data flow, green arrows represent the information flow during model learning, and dashed green arrows denote iterative feedback for model refinement

Deep learning has become a central analytical paradigm in single-cell research, offering flexible solutions to handle the sparsity, noise, and high dimensionality inherent in omics data, which is used in important steps of single-cell analysis. Current deep learning methods for single-cell analysis are primarily built upon four representative architectures: autoencoders (AEs) [170], graph neural networks (GNNs) [171], transformer-based models [172], and foundation models [173] (Fig. 4). Autoencoders compress high-dimensional gene expression profiles into compact latent representations and reconstruct the original data, making them particularly suitable for denoising, dimensionality reduction, and batch correction. GNNs explicitly model relationships among cells or genes as graphs, enabling effective learning of cellular interactions and regulatory structures. Transformer-based models employ self-attention mechanisms to capture long-range dependencies among genes and cells, providing greater scalability and transferability across datasets. Building upon Transformer architectures, foundation models are pretrained on large-scale single-cell datasets to learn generalizable biological representations that can be adapted to diverse downstream tasks through fine-tuning or prompting. These architectures differ substantially in their underlying learning strategies and are therefore suited to different analytical applications, which are discussed below.

#### Denoising and imputation

Single-cell sequencing technologies remain limited by technical noise, including amplification bias, cell cycle effects, library size differences, and particularly low RNA capture rates that lead to dropout events (false zeros) [174]. Autoencoders (AEs) have been widely applied to denoising and imputation tasks [175]. *AutoImpute* introduced an overcomplete AE that reconstructs expression matrices by learning high-dimensional latent representations [176]. *DCA* extended this by modeling zero-inflated negative binomial distributions, enabling scalability to millions of cells [175]. *DeepImpute* adopted a divide-and-conquer strategy with multiple sub-networks, outperforming DCA in imputation accuracy [177]. In parallel, *scVI* applied a hierarchical Bayesian framework parameterized by DNNs, effectively imputing dropouts while maintaining biological signal integrity [69]. Together, these models highlight how deep learning provides powerful and scalable strategies for denoising sparse scRNA-seq data.

#### Batch effect correction

Large-scale single-cell datasets inevitably suffer from batch effects caused by technical artifacts or uncontrolled biological variables. Unlike traditional linear approaches (e.g., Harmony, CCA) [50, 67], deep learning methods capture nonlinear batch effects, scale efficiently to large datasets, and integrate across modalities. *MMD-ResNets* demonstrated the use of residual networks combined with maximum mean discrepancy to align distributions while preserving biological signals, successfully applied to scRNA-seq and mass cytometry data [178]. AE-based approaches have also flourished. *BEENE* generated nonlinear embeddings that retained biological variability while outperforming Harmony and MNN Correct [179]. *scMEDAL* introduced a mixed-effects AE to separate batch-invariant from batch-specific signals, enabling retrospective prediction of expression profiles across batches [180]. Additionally, methods such as *DESC* and *scDML* jointly optimized clustering and batch correction, preserving rare cell types during integration [181, 182]. Overall, deep learning has expanded the toolkit for batch correction, providing nonlinear, scalable, and biologically faithful alternatives to classical approaches.

#### Cell-type annotation

Following QC and batch correction, clustering and annotation define cellular identities and states. Traditional clustering methods (e.g., k-means, Louvain, Leiden) often fail to capture nonlinear dependencies or integrate multimodal data [183, 184]. Deep learning addresses these challenges by learning more informative embeddings. *scDeepCluster* jointly optimized feature representation and clustering, outperforming conventional methods across metrics and scaling linearly with sample size [185]. Building on this, AE-based methods such as *scANVI*, *scVI*, and *scziDesk* improved cluster-friendly embeddings [69, 186, 187]. *scAnCluster* further integrated marker information into a supervised framework, supporting intra- and inter-dataset annotation and detecting novel cell types [188]. Beyond AE-based methods, GNNs have enabled reference-free annotation. *scDeepSort* applied a weighted GNN to annotate > 760 k cells across 56 human and 32 mouse tissues [189], while *scGCN* enhanced cross-dataset transfer through graph convolution [190]. More recently, transformer-based models such as *scFormer* and *scGraphformer* introduced self-attention and graph–transformer hybrids, jointly learning cell–gene embeddings and capturing subtle relational patterns [191, 192]. These advances illustrate the transition from AE-based clustering toward GNNs and transformers, offering more accurate, scalable, and interpretable cell-type annotation.

#### Downstream biological analysis

Deep learning also extends to downstream tasks, including trajectory inference and cell–cell interaction (CCI) analysis. For CCIs, *DeepCCI* clustered cells based on complex gene expression patterns to infer biologically meaningful interactions [193], while *DeepTalk* applied graph attention networks to capture cell–cell connections at both single-cell and spatial levels [194]. For trajectory inference, *VITAE* combined variational AEs with hierarchical mixture models to jointly infer trajectories and integrate heterogeneous datasets [195]. *DeepVelo* leveraged deep GCNs to predict RNA velocity without predefined kinetic assumptions, accommodating complex branching lineages [196].

Overall, the suitability of deep learning models for single-cell analysis largely depends on their underlying architecture and the analytical task. Autoencoder-based models are computationally efficient and well suited to denoising, dimensionality reduction, and batch correction, although their latent representations can be difficult to interpret. Graph neural networks explicitly model relationships among cells or genes, making them advantageous for cell-type annotation, regulatory-network inference, and cell–cell communication analysis, but their performance is sensitive to graph construction. Transformer-based models can capture long-range gene–gene dependencies and offer greater scalability and transferability, yet they generally require larger training datasets and substantially greater computational resources. Consequently, autoencoder-based models remain practical for well-defined preprocessing and representation-learning tasks, whereas graph- and Transformer-based approaches are more appropriate for relational or large-scale integrative analyses.

### Foundation models for knowledge integration

Over the past decade, deep learning architectures—including feed-forward, convolutional, recurrent, and graph neural networks—have become integral to single-cell analysis, enabling denoising, clustering, annotation, and multimodal integration [197]. The introduction of Transformer models represented a major leap forward by capturing long-range dependencies in high-dimensional biological data [172]. Building upon this foundation, large language models (LLMs) scale Transformers through massive pretraining to achieve broad generalization [198]. Inspired by this paradigm, emerging LLM-based models in single-cell biology are now driving advances in automated annotation, multimodal integration, and predictive modeling, thus extending deep learning from task-specific applications to universal foundation models [197].

Among these, scFoundation is a 100-million-parameter model pretrained on over 50 million scRNA-seq profiles [199]. It employs an asymmetric Transformer architecture with read-depth–aware pretraining to capture gene–gene co-expression while accounting for technical variation. Demonstrating broad applicability, scFoundation supports diverse downstream tasks including clustering, cell type annotation, drug response prediction, perturbation analysis, and gene regulatory network inference, underscoring its scalability and versatility. In parallel, scGPT introduced a generative pretraining workflow tailored for non-sequential omics data [76]. Trained on over 33 million cells, it jointly learns cell and gene embeddings and follows a “pretrain universally, fine-tune on demand” paradigm. This framework achieved state-of-the-art performance in annotation, batch correction, perturbation prediction, and multi-omics integration. Notably, scaling analyses revealed that larger pretraining datasets yield more informative embeddings, highlighting the potential for continuous performance gains as single-cell resources expand. Complementing these models, Geneformer leveraged transfer learning and Transformer-based attention mechanisms to learn gene regulatory networks from large-scale transcriptomic data [77]. By capturing context-specific gene–gene interactions, Geneformer enables accurate modeling of regulatory dynamics even in settings with limited data, such as rare diseases, and shows promise for applications ranging from cell type and gene function prediction to network-correcting drug discovery.

Large-scale pretraining distinguishes foundation models from conventional task-specific deep learning approaches by enabling transferable representations that can be adapted to multiple downstream applications with limited additional training. This broader applicability, however, comes with greater computational demands, reduced interpretability, and potential sensitivity to biases in the pretraining data. Among representative models, scFoundation emphasizes large-scale gene-expression representation and broad task coverage [199], scGPT provides a flexible generative framework for annotation, integration, and perturbation analysis [76], whereas Geneformer is particularly suited to modeling context-dependent gene regulatory relationships [77]. Because their relative performance varies across datasets and tasks, current evidence does not support a universally superior model. Model selection should therefore consider the intended application, computational resources, availability of pretrained weights, and the similarity between the target dataset and the pretraining corpus. In addition, the performance of foundation models may be influenced by biases in the pretraining data, including uneven representation of tissues, developmental stages, species, and disease conditions. Consequently, predictions generated by these models should be interpreted cautiously and, whenever possible, validated using independent datasets and experimental approaches before biological or translational applications [200]. Most currently available foundation models have been pretrained predominantly on human datasets. Extending these models to farmed animals will require either large-scale species-specific pretraining datasets or transfer-learning strategies capable of accommodating evolutionary divergence in gene regulation and cellular composition.

### AI-driven perturbation and predictive modeling

While foundation models primarily learn transferable biological representations from observed data, a new generation of AI models has shifted the focus from representation learning toward predictive modeling of cellular perturbations [201, 202]. Rather than simply characterizing existing cellular states, these approaches aim to forecast transcriptional responses to genetic variation, CRISPR perturbations, drug treatments, and environmental stimuli, enabling in silico experimentation without requiring every perturbation to be experimentally performed. This transition marks an important conceptual advance, extending AI from descriptive analysis to predictive simulation of cellular behavior.

One major direction is the prediction of cellular responses to experimental perturbations. Deep generative models leverage latent representations of cellular states to infer how gene expression changes following external perturbations. For example, scGen introduced a variational autoencoder-based framework that models perturbation vectors in latent space, allowing accurate prediction of transcriptional responses under previously unseen conditions across different cell types and species [203]. Building upon this concept, CPA (Compositional Perturbation Autoencoder) further incorporated drug dosage, cell identity, and combinatorial perturbations into a unified framework, enabling prediction of complex perturbation effects beyond those directly observed experimentally [204]. More recently, GEARS integrated graph neural networks with prior knowledge of gene interactions to predict the consequences of single and combinatorial CRISPR perturbations, providing a scalable framework for interpreting large-scale perturbation screening data [205]. Together, these models substantially reduce the experimental burden of perturbation studies while enabling systematic exploration of cellular responses across diverse biological contexts. Although these models provide an efficient framework for hypothesis generation, experimentally validating predicted perturbation effects remains essential, particularly when extrapolating to unseen perturbations or new biological systems. In addition, because perturbation prediction models learn general relationships between molecular perturbations and cellular responses, their analytical framework is readily transferable to farmed animals. However, broader application will require substantially more perturbation datasets across farmed animal tissues and cell types.

Beyond experimental perturbations, AI models are increasingly being developed to predict the functional consequences of naturally occurring genetic variation. Rather than directly measuring the regulatory effects of millions of variants experimentally, these approaches infer how sequence variation reshapes gene regulation and cellular phenotypes computationally. Transformer-based foundation models, including Geneformer and scGPT, have demonstrated the capacity to capture context-dependent gene regulatory programs and predict transcriptional responses under perturbed conditions [76, 77]. In parallel, sequence-based deep learning models capable of predicting regulatory variant effects from genomic DNA have further expanded the ability to prioritize functional non-coding variants and interpret genetic mechanisms underlying complex traits and diseases [206]. Collectively, these advances establish a computational framework for linking genetic variation with cell-state transitions and regulatory network dynamics at single-cell resolution.

By enabling in silico prediction of cellular responses, these models can reduce the experimental burden of perturbation studies, although their suitability differs according to the perturbation setting. scGen provides a relatively simple latent-space framework for predicting responses across cell types or conditions, but it may be less effective for modeling complex combinatorial effects [203]. CPA explicitly separates perturbation, dosage, and cellular-context effects, making it particularly suitable for dose-dependent and combinatorial drug-response prediction [204]. GEARS further incorporates gene-interaction networks and is therefore advantageous for predicting single and combinatorial genetic perturbations, although its performance depends on the quality and transferability of the underlying graph [205]. The optimal model consequently depends on whether the primary objective is cross-condition prediction, pharmacological response modeling, or genetic perturbation analysis.

Looking beyond individual perturbation models, recent efforts have proposed the concept of AI Virtual Cells (AIVCs), which aim to integrate Transformer architectures with large-scale single-cell multi-omics datasets to simulate molecular, cellular, and tissue behaviors across diverse biological contexts [201, 202, 207]. As multi-scale and multimodal neural network systems, AIVCs seek to (1) generate universal representations of biological states across species, modalities, developmental stages, and environmental conditions; (2) predict cellular functions, behaviors, and dynamic responses while revealing their underlying regulatory mechanisms; and (3) enable in silico experimentation to test biological hypotheses and guide experimental design. Likewise, AI-based virtual cells and tissues should therefore be viewed as complementary tools for generating and prioritizing hypotheses rather than as substitutes for experimental validation [208]. In farmed animals, AI virtual cells may ultimately facilitate precision breeding by predicting the cellular consequences of naturally occurring genetic variants, although achieving this goal will require substantially richer multimodal datasets than are currently available.

## Application of single-cell data in human and farmed animals

As single-cell datasets expand, the field is shifting from descriptive atlases toward biological discovery and translational application. Single-cell technologies can resolve cell-type specific mechanisms underlying complex traits and diseases and facilitate therapeutic target identification, drug-response prediction, and precision medicine. These applications illustrate how cell-resolved molecular information can bridge genetic regulation, organismal phenotypes, and clinical outcomes.

### Understanding complex traits or diseases using single-cell data

With methodological advances and the publication of large-scale datasets, single-cell sequencing technologies provide a powerful framework to connect molecular phenotypes and genetic variants with cellular phenotypes, thereby improving the understanding of trait and disease-associated mechanisms. Four complementary analytical strategies are commonly used to connect single-cell molecular profiles with complex traits and diseases. Cell-type enrichment analyses prioritize the cellular populations in which trait-associated variants are concentrated. Locus-level integration methods, including colocalization and summary-data-based Mendelian randomization, link GWAS signals to candidate molecular mechanisms. Transcriptome-wide association studies further evaluate trait associations with genetically predicted gene expression across the genome, whereas predictive models capture nonlinear relationships and estimate disease-associated cellular states or genetic effects. These approaches operate at different levels of inference, ranging from cell-type prioritization and locus interpretation to gene-level association and phenotype prediction.

Cell-type enrichment analysis is used in identifying causal cell types related to diseases or traits by integrating GWAS summary data with annotated cell atlases. In 2017, Calderon et al. [209] introduced RolyPoly, a variance-component regression model that links GWAS variants to gene expression across tissues or cell types, thereby identifying trait-relevant cell-types under polygenic background. Subsequent frameworks expanded on this principle. MAGMA, a leading gene set analysis tool, evaluates enrichment of GWAS associations in cell-type specific genes by performing gene-level statistics (accounting for LD) and regressing them against normalized expression specificity scores. Building on MAGMA, Skene et al. [210] demonstrated that schizophrenia risk variants map predominantly to pyramidal cells, MSNs and interneurons, but not to progenitors or glia. In parallel, Finucane et al. [211] developed LDSC-SEG, applying stratified LD score regression to specifically expressed genes across expression and chromatin datasets, thereby revealing polygenic risk to disease-relevant tissues and cell-types such as brain and immune cells. To extend cross-study robustness, Watanabe et al. [212] developed FUMA, a web-based platform using conditional analyses across multiple scRNA-seq datasets to identify consistent and independent cell type-trait associations, including cardiovascular, cognitive and psychiatric traits. More recently, Ma et al. developed scPagwas, which integrates scRNA-seq and GWAS via pathway-level polygenic regression. By mapping GWAS signals to pathway activation scores rather than single-gene specificity, scPagwas enhances statistical power, reduces confounding and identifies trait-relevant subpopulations, exemplified by understanding into naïve CD8⁺ T cells in COVID-19 severity and microglia/OPCs in Alzheimer’s disease risk [213]. In farmed animals, Gao et al. [118] performed enrichment between DEGs from seven cell-types in PBMC and 45 cattle traits and found the association of immune cells with health, milk production and body conformation traits. Together, these methods highlight the evolution of cell-type enrichment strategies from gene-level specificity toward pathway-level integration, improving the resolution and robustness of linking GWAS signals to cellular phenotypes.

Although these approaches can highlight statistical associations between specific cell types and complex traits or diseases, they cannot directly pinpoint the causal genes or variants. Moreover, their applicability is often constrained by different contexts. To overcome these limitations, we turn to introducing the population-level analyses by integrating molQTL with GWAS data, which supports more direct inference of trait/disease-relevant genes and their causal variants at cellular level. Colocalization is one of the most widely used approaches for this purpose, as it evaluates whether a molQTL and a GWAS association within the same genomic region are likely to be driven by a shared causal variant [214]. Using this strategy, Yazar et al. [159] identified 19% *cis*-eQTL colocalized with causal variants in seven autoimmune diseases based on 26,597 independent *cis*-eQTL in 14 cell-types. Of the colocalized causal loci, 38.4% are outside the Major Histocompatibility Complex (MHC) region and exhibit highly cell-specific effects. Another study about cell-type response eQTL (reQTL) defined 1,955 colocalized eGenes with 83 diseases and complex traits based on 24 cellular conditions of human induced Pluripotent Stem Cells (iPSC)-derived macrophages. This study identified 38.6% novel colocalized signals between reQTL and GWAS loci, which were not found in the Genotype-Tissue Expression (GTEx) project [215]. Additionally, based on interaction analysis, Kim-Hellmuth et al. [12] identified that 34% of GWAS loci in 87 human complex traits can be specifically explained by ieGenes and isGenes in seven cell-types which were not found in bulk level. Of them, 1,370 (10.3%) cell-type ieQTL and 89 (3.7%) isQTL colocalized with at least one GWAS trait, suggesting that cell-type interaction QTL captured novel molecular regulation for GWAS loci that are missed by bulk QTL. As for other single-cell molecular QTL, Zeng et al. [216] performed colocalization analysis to identify shared regulatory signals across chromatin accessibility (ca), gene expression and GWAS loci of neuropsychiatric and neurodegenerative diseases, uncovering 98 colocalized signals and 53 caQTL-GWAS pairs without an eQTL signal, highlighting the advantage of caQTL in directly linking noncoding variants to regulatory elements beyond gene expression. To further refine candidate regulatory variants within associated loci, statistical fine-mapping analysis is commonly performed alongside colocalization analyses. Fine-mapping estimates the posterior probability that individual variants are causal by jointly modeling linkage disequilibrium and association signals, thereby reducing large association intervals to a small set of credible variants [217]. In practice, integrating fine-mapping with cell-type specific molQTL and functional genomic annotations can substantially improve the prioritization of candidate causal variants for downstream experimental validation. For example, Li et al. [13] provides a list of 898 candidate cell-type eQTL for 44 cattle complex traits by combining colocalization and fine mapping analysis. In contrast to colocalization and fine-mapping, which primarily evaluate shared association signals and prioritize candidate variants at specific loci, summary-data-based Mendelian randomization (SMR) tests whether the genetically regulated component of a molecular phenotype is associated with a complex trait by using molecular QTL as instrumental variables [218]. SMR is therefore particularly useful for prioritizing genes or molecular phenotypes that may contribute to trait variation. For example, Liu et al. [219] used SMR to identify 17 genes across nine immune cell types associated with overall breast cancer or its subtypes. In another study, SMR was combined with colocalization to prioritize 28 candidate genes associated with Alzheimer’s disease risk using five GWAS datasets and two cell type-specific eQTL datasets derived from single-cell sequencing of Alzheimer’s disease brain samples [220]. These findings illustrate how SMR can prioritize molecular phenotypes that may mediate trait-associated genetic effects, although further colocalization and functional validation are required for causal interpretation. However, a significant SMR association may result either from a shared causal variant or from distinct causal variants in linkage disequilibrium. The heterogeneity in dependent instruments (HEIDI) test is commonly used to assess whether the observed association is consistent with a single shared signal rather than linkage between separate variants [221].

Transcriptome-wide association studies (TWAS) extend molQTL–GWAS integration to the gene level by testing whether genetically predicted gene expression is associated with a trait across the genome [222]. Their main advantage is the ability to improve statistical power and prioritize candidate genes by aggregating the effects of multiple *cis*-regulatory variants into a single expression-prediction model. However, TWAS associations may reflect linkage disequilibrium, correlated expression among nearby genes, or shared regulatory architecture and therefore do not necessarily identify the causal gene. Using cell-type specific expression reference panels, Wang et al. developed EXPRESSO, a cell-type-specific TWAS framework, and identified 958 gene–trait associations across 14 autoimmune diseases, including 492 associations that were missed by conventional whole-blood TWAS [223]. Cell-type specific TWAS can further refine these associations by identifying the cellular contexts in which genetically regulated expression is most relevant to the phenotype. Together, these three approaches provide complementary but non-equivalent evidence. Colocalization asks whether molecular and trait association signals share a causal variant, SMR tests whether genetically regulated molecular phenotypes are associated with the trait, and TWAS evaluates trait associations with genetically predicted gene expression at the gene level. Colocalization generally offers more direct locus-level evidence, whereas SMR and TWAS can achieve greater power for gene prioritization by aggregating information across variants. However, both SMR and TWAS remain susceptible to linkage disequilibrium and horizontal pleiotropy, and none of these methods alone is sufficient to establish causality.

Although enrichment analyses, molQTL colocalization, SMR and TWAS provide valuable insights into trait-cell type associations, their resolution is often constrained by linkage disequilibrium (LD) and limited statistical power. In contrast, deep learning-based approaches can learn high-dimensional and nonlinear dependencies directly from large-scale single-cell data, overcoming LD-related confounding and supporting more accurate prediction of trait-cell type associations in complex biological contexts. Recently, several methods have been developed to predict associations of cell types with diseases or complex traits. For example, scPRS leverages graph neural networks to construct single-cell-resolved polygenic risk scores from chromatin accessibility profiles, outperforming traditional PRS in disease risk prediction and fine-mapping causal variants and cell types across multiple diseases [224]. scPrediXcan extends the Transcriptome-Wide Association Study (TWAS) framework to the single-cell level by combining deep learning-based expression prediction (ctPred/ℓ-ctPred) with GWAS data, thereby identifying causal genes and uncovering cell-type specific regulatory mechanisms with higher resolution than traditional TWAS [206]. In parallel, scIDST introduces a weakly supervised deep learning strategy to infer disease progression states of individual cells from patient-derived scRNA-seq, allowing the detection of disease-relevant gene dysregulation and comorbid cell populations that escape conventional case–control analyses [225]. Together, these approaches highlight the power of deep learning to overcome LD-related limitations, enhance trait-to-cell type mapping and provide mechanistic insights into disease biology at single-cell resolution.

Overall, no single approach provides sufficient evidence to fully resolve the genetic mechanisms underlying complex traits, and these methods are therefore most informative when integrated as complementary lines of evidence. Concordant evidence across cell-type prioritization, gene- and locus-level association, colocalization, and statistical fine-mapping can strengthen the prioritization of candidate genes and variants, while predictive models provide complementary information on nonlinear molecular patterns. Nevertheless, statistical or predictive evidence alone does not establish causality, and functional annotation and experimental perturbation remain important for validating the direction and mechanisms of inferred regulatory effects. Many of these analytical frameworks, initially developed and extensively applied in human genetics, have also been adopted in farmed animals to identify trait-relevant cell types, candidate genes, and regulatory variants underlying economically important traits [3–5, 7]. As livestock single-cell atlases and molecular QTL resources continue to expand, their integration with larger population cohorts and increasingly diverse molecular phenotypes should further improve the resolution and biological interpretation of complex-trait genetics.

### Drug discovery and precision medicine

Having illustrated how single-cell technologies reveal the cellular mechanisms underlying complex traits and diseases, the next challenge is to translate these insights into therapeutic interventions. Traditional drug development remains costly, time-consuming, and prone to high failure rates, partly because bulk-level measurements obscure heterogeneous responses across cell populations [226]. By resolving drug-induced molecular changes at cellular resolution, single-cell sequencing can identify therapeutic targets, characterize treatment-responsive cell states, and reveal cell-type specific mechanisms of drug action [227]. Current applications can be broadly classified into three complementary strategies: experimental perturbation screens that provide direct functional evidence for therapeutic targets, transcriptomic signature-matching approaches that prioritize candidate compounds, and AI-based models that predict responses to previously untested perturbations. These strategies differ in their evidential strength, scalability, and roles at different stages of drug development.

Early efforts focused on perturbation-based technologies that directly coupled CRISPR-mediated genetic perturbations with single-cell transcriptomics. Representative methods such as Perturb-seq, CRISP-seq, and CROP-seq enabled high-throughput characterization of gene function by measuring transcriptional responses following targeted gene perturbations at single-cell resolution [228–230]. These approaches identified disease-relevant gene networks, regulatory pathways, and cellular states associated with therapeutic responses, establishing an experimental framework for functional target discovery and mechanism-of-action studies. Compared with computational prediction methods, pooled CRISPR–single-cell screening provides direct functional evidence for target validation and mechanism-of-action studies. However, these approaches remain experimentally demanding, requiring tractable cellular systems, high sequencing depth, and substantial resources, which currently limit their scalability for large-scale therapeutic screening [230]. Consequently, perturbation screens are most effective after candidate genes have been prioritized through genetic or computational analyses rather than as the initial discovery strategy.

Building upon these experimental platforms, computational approaches have substantially expanded the application of single-cell data in drug discovery. By integrating disease-associated transcriptional signatures with large-scale perturbation resources such as the Connectivity Map (CMap) and LINCS, these methods enable systematic drug repurposing and prediction of therapeutic responses [231, 232]. For example, Musa et al. integrated LINCS-derived drug-induced transcriptional profiles to construct drug association networks, revealing groups of drugs with shared transcriptional responses and mechanisms of action, thereby facilitating drug repurposing and therapeutic target prioritization [231]. To further streamline this process, Hsieh et al. developed scDrug, an integrated computational workflow that combines single-cell analysis, tumor subpopulation identification, functional annotation, and drug response prediction within a unified framework, facilitating systematic prioritization of candidate therapeutics [233]. Unlike perturbation screens, signature-matching approaches prioritize therapeutic compounds by comparing disease-associated transcriptional profiles with reference drug-response signatures, making them particularly suitable for drug repurposing [234]. Their major advantage lies in the ability to rapidly evaluate thousands of compounds without additional experiments. However, prediction accuracy depends strongly on the availability and relevance of reference datasets, and candidate drugs generally require subsequent experimental validation [235]. These approaches are therefore especially valuable during the early stages of therapeutic discovery, when rapid candidate prioritization is more important than definitive mechanistic validation.

More recently, AI-based predictive models have further accelerated the translation of single-cell data into therapeutic discovery. Rather than relying exclusively on experimentally measured perturbation datasets, deep learning and foundation models enable computational prediction of cellular responses under previously unseen conditions, substantially reducing experimental costs while improving scalability. As discussed in Sect. "Artificial intelligence and foundation models in single-cell analysis", predictive models such as scGen, scFoundation, scGPT, and Geneformer provide transferable representations that support virtual perturbation screening, drug sensitivity prediction, therapeutic target prioritization, and rational design of drug combinations [76, 77, 199, 203]. Together, these advances are transforming single-cell genomics from a descriptive technology into a predictive platform that supports drug repurposing, precision medicine, and the development of more effective personalized therapies. Compared with conventional signature-matching approaches, AI models can predict responses to previously unobserved perturbations, treatment combinations, or cellular contexts, substantially expanding the search space for therapeutic discovery. Nevertheless, their performance depends on the diversity and quality of training data, and predictions should be regarded as hypothesis-generating rather than definitive evidence of therapeutic efficacy.

Rather than representing competing strategies, these approaches are most effective when integrated into a sequential drug-discovery workflow. Genetic analyses and computational models can first prioritize candidate targets and compounds, followed by targeted perturbation screens to evaluate mechanisms of action and validate therapeutic effects. Such iterative integration of prediction and experimental validation is likely to accelerate both drug repurposing and precision medicine while reducing experimental cost and improving biological interpretability [236]. Beyond human precision medicine, these analytical strategies are also expected to facilitate veterinary drug discovery and precision breeding by enabling cell-type specific target prioritization and prediction of treatment responses in farmed animal species.

Overall, compared with human studies, current farmed animal applications remain concentrated in atlas construction and trait interpretation, whereas population-scale single-cell genetics, multimodal molecular QTL mapping, and AI-assisted functional prediction are still in their infancy. Future progress will rely on expanding species-specific reference atlases, increasing population-scale datasets, integrating multiple omics layers, and establishing experimental systems for functional validation.

## Conclusion and future perspectives

### Toward a closed loop: from genotypes to phenotypes

Single-cell technologies have transformed our ability to map genetic variants to molecular phenotypes, cell types, and regulatory programs, offering unprecedented resolution into the architecture of complex traits and diseases. From enrichment analyses and molecular QTL colocalization to deep learning and large language models (LLMs), the methodological landscape has rapidly evolved to address limitations of linkage disequilibrium, data sparsity, and nonlinear regulation. Moving forward, the integration of single-cell multi-omics, perturbation screens, and AI-powered predictive models promises to build a closed-loop framework linking genotype to phenotype. Such a framework will not only illuminate fundamental biological principles but also enable rational strategies for therapeutic intervention and genomic selection.

### Toward population-scale single-cell genomics in farmed animals

Although single-cell technologies have rapidly advanced human genomics, their application in farmed animals remains limited. Most functional genomics resources in domesticated animals are still generated from bulk tissues, making it difficult to resolve the cell-type specific regulatory mechanisms through which genetic variants influence complex traits. Computational deconvolution provides a practical alternative by estimating cell-type composition and expression profiles from bulk RNA-seq data [139]. However, its performance depends heavily on the quality and completeness of reference single-cell atlases and is often challenged by rare cell types, dynamic cellular states, and condition-specific transcriptional programs [149].

Looking ahead, establishing population-scale single-cell datasets with matched genotype information will be a critical priority for farmed animal research. Such resources will enable the direct identification of cell-type specific molecular QTL and provide a more comprehensive understanding of how genetic variation shapes complex traits at cellular resolution. Combined with AI-driven predictive models and genomic selection, population-scale single-cell genomics has the potential to transform functional genomics and accelerate precision breeding in agricultural species.

### AI-powered precision medicine and farmed animal improvement

Artificial intelligence is poised to transform farmed animal functional genomics by leveraging single-cell data to dissect the cellular mechanisms underlying economically important traits, predict the effects of genetic perturbations, and guide genomic selection. Deep learning methods such as scGen and variational autoencoders have demonstrated robust prediction of cellular perturbation outcomes, while foundation models such as scFoundation and scGPT further extend these capabilities by providing universal and generalizable representations applicable across diverse downstream tasks. These advances also create new opportunities for veterinary medicine, enabling the prediction of drug responses, identification of therapeutic targets, and rational design of veterinary drugs and intervention strategies for infectious, metabolic, and production-related diseases in farmed animals. Beyond animal science, the same AI-powered frameworks are expected to redefine precision medicine in humans by facilitating disease mechanism dissection, drug response prediction, rational design of therapeutic combinations, and personalized treatment strategies at cellular resolution. Collectively, AI-driven single-cell frameworks provide a unified paradigm for predictive biology, promising transformative impacts on veterinary medicine, human healthcare, sustainable farmed animal improvement, and next-generation precision therapeutics [76, 199, 203].

### Remaining challenges and collaborative needs

Despite these advances, significant challenges remain. Current single-cell studies are often limited by data sparsity, technical variability, and incomplete coverage across tissues, cell types, and species. Standardization of experimental protocols and computational pipelines is urgently needed to enable reproducibility and large-scale integration. Moreover, the generalization of AI and LLM-based models across species and contexts requires richer training datasets, advanced transfer learning strategies, and careful benchmarking to avoid overfitting or bias.

As the field progresses from task-specific models toward foundation models and even AIVCs, these challenges become amplified and more complex [201, 202, 207]. Unlike conventional applications, AIVCs aim to provide universal representations and predictive simulations across scales, modalities, and species, making issues such as capability definition and benchmarking particularly critical. Ensuring self-consistency across biological contexts, from molecular interactions to tissue-level organization, remains a formidable hurdle, especially when accounting for stochasticity and species differences. In addition, the trade-off between predictive performance and interpretability must be carefully managed to ensure actionable biological insights. Finally, the success of AIVCs will depend on building collaborative infrastructures, promoting ethical and responsible data use, and generating diverse, large-scale datasets that capture the full spectrum of biological complexity.

Ultimately, realizing the full potential of single-cell technologies and AIVCs will require close interdisciplinary collaboration, bridging genomics, computational biology, artificial intelligence, medicine, and agriculture. Collaborative efforts in data sharing, open-source tool development, and cross-disciplinary partnerships will be essential to translate these innovations into real-world clinical and agricultural applications.

Looking forward, continued methodological advances will likely be driven by human studies, whereas one of the major opportunities lies in transferring and refining these computational frameworks to farmed animal populations. Together with the rapid expansion of farmed animal single-cell resources, such translation is expected to accelerate both fundamental biological discovery and practical applications in animal breeding and veterinary medicine.

## Data Availability

No datasets were generated or analysed during the current study.

## Abbreviations

AE: Autoencoder
AI: Artificial intelligence
AIDA: Asian Immune Diversity Atlas
AIVC: AI Virtual Cell
APA: Alternative polyadenylation
ATAC: Assay for transposase-accessible chromatin
caQTL: Chromatin accessibility quantitative trait locus
CCA: Canonical correlation analysis
CCI: Cell–cell interaction
cDNA: Complementary DNA
CRISPR: Clustered regularly interspaced short palindromic repeats
DE: Differential expression
DEG: Differentially expressed gene
DNN: Deep neural network
EC: Endothelial cell
eQTL: Expression quantitative trait locus
FarmGTEx: Farm Animal Genotype-Tissue Expression
GNN: Graph neural network
GRN: Gene regulatory network
GSEA: Gene set enrichment analysis
GTEx: Genotype-Tissue Expression
GWAS: Genome-wide association study
HCA: Human Cell Atlas
HCL: Human Cell Landscape
HDCA: Human Developmental Cell Atlas
HEIDI: Heterogeneity in dependent instruments
ieQTL: Interaction expression quantitative traitlocus
iPSC: Induced pluripotent stem cell
isQTL: Interaction splicing quantitative trait locus
LD: Linkage disequilibrium
LM: Large language model
LM: Linear model
LMM: Linear mixed model
lncRNA: Long non-coding RNA
LPS: Lipopolysaccharide
MCDA: Mouse Cell Differentiation Atlas
meQTL: Methylation quantitative trait locus
MHC: Major histocompatibility complex
molQTL: Molecular quantitative trait locus
mRNA: Messenger RNA
NGS: Next-generation sequencing
NHPCA: Non-human Primate Cell Atlas
PBMC: Peripheral blood mononuclear cell
PCA: Principal component analysis
PGC: Primordial germ cell
PRS: Polygenic risk score
QC: Quality control
QTL: Quantitative trait locus
reQTL: Response expression quantitative trait locus
RNA-seq: RNA sequencing
scATAC-seq: Single-cell assay for transposase-accessible chromatin using sequencing
scRNA-seq: Single-cell RNA sequencing
SMR: Summary-data-based Mendelian randomization
sQTL: Splicing quantitative trait locus
TAD: Topologically associating domain
TF: Transcription factor
t-SNE: t-distributed stochastic neighbor embedding
TWAS: Transcriptome-wide association study
UMAP: Uniform manifold approximation and projection
UMI: Unique molecular identifier
UTR: Untranslated region
